# Raman Spectroscopy Reveals Phase Separation in Imine-Based Covalent Adaptable Networks

**DOI:** 10.1021/acs.macromol.2c01595

**Published:** 2022-11-30

**Authors:** Sybren K. Schoustra, Martijn H. P. de Heer Kloots, Joris Posthuma, Daphne van Doorn, Joshua A. Dijksman, Maarten M. J. Smulders

**Affiliations:** † Laboratory of Organic Chemistry, Wageningen University, Stippeneng 4, 6708 WE Wageningen, The Netherlands; ‡ Department of Physical Chemistry and Soft Matter, Wageningen University, Stippeneng 4, 6708 WE Wageningen, The Netherlands

## Abstract

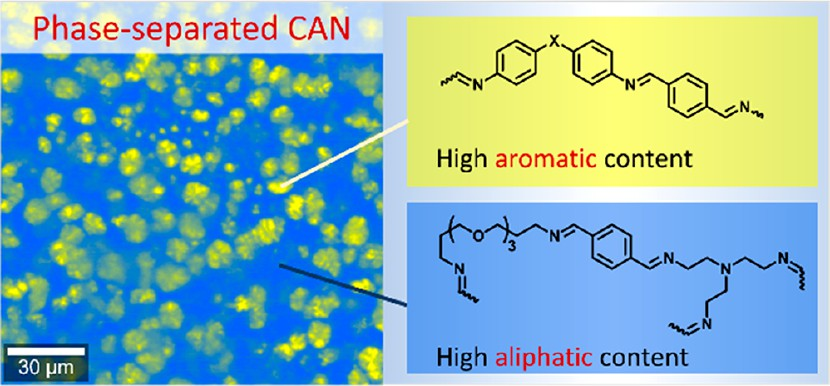

The introduction of dynamic covalent bonds into cross-linked polymer networks enables the development of strong and tough materials that can still be recycled or repurposed in a sustainable manner. To achieve the full potential of these covalent adaptable networks (CANs), it is essential to understand—and control—the underlying chemistry and physics of the dynamic covalent bonds that undergo bond exchange reactions in the network. In particular, understanding the structure of the network architecture that is assembled dynamically in a CAN is crucial, as exchange processes within this network will dictate the dynamic-mechanical material properties. In this context, the introduction of phase separation in different network hierarchies has been proposed as a useful handle to control or improve the material properties of CANs. Here we report—for the first time—how Raman confocal microscopy can be used to visualize phase separation in imine-based CANs on the scale of several micrometers. Independently, atomic force microscopy (AFM) confirmed the phase-separated domains inside the polymer. Remarkably, the materials were found to undergo phase separation despite being built up from miscible monomers, which arguably should yield homogeneous materials. We found that the phase separation not only affected the appearance of the material but—more notably—also had a noticeable effect on the thermal-mechanical properties of the material: CANs (of equal aliphatic/aromatic monomer composition) that displayed phase separation had both a higher crossover temperature (*T*_cross_, where tan(δ) = 1, and where the material transits from a rubbery to a viscous state) and an increased elastic modulus (*G*′). By modifying the CAN architecture, we were able to either suppress or enhance the phase separation, and we propose that the phase separation is driven by favorable π–π interactions between the aromatic components. Our work further shows the importance of phase separation in CANs, including in networks built from miscible components, and provides a handle to control the dynamic material properties. Moreover, our work underlines the suitability of Raman imaging as a method to visualize phase separation in CANs.

## Introduction

Thermosets have a permanent cross-linked polymer network structure that gives them high material strength and resistance against many environments, which makes them suitable for many industrial applications. However, due to this permanent network structure, thermosets cannot be easily recycled or repurposed once they have been produced. Therefore, the use of current thermosetting materials is not sustainable. To overcome this problem, dynamic covalent bonds can be introduced within the polymeric structure to construct covalent adaptable networks (CANs) that enable reprocessability and recyclability.^1,2^ These dynamic covalent bonds can be just as strong as their nonreversible covalent counterparts and as such do not decrease the strength of the material. What makes dynamic covalent bonds so interesting is that they can perform bond-exchange reactions.^3,4^ This bond exchange requires some sort of activation, which is generally achieved with heat, but other triggers (e.g., light or pH) are known as well.^5^ In some cases a catalyst may be required that can be either external or internal.^6−9^ The ability to exchange covalent bonds in the polymeric network of thermosets enables the material to flow and, as such, facilitates (re)processability.

The potential of CANs to replace or improve classical thermosets has inspired many researchers to develop and study the underlying chemistry and physics.^10−14^ Many of these studies involved application of different dynamic chemistries, each with its characteristic properties based on the nature of the bond-exchange reaction.^15−19^ Examples of different dynamic chemistries include transesterification reactions,^20−22^ disulfide exchange,^23−27^ Diels–Alder reactions,^28−30^ (vinylogous) urethane exchange,^31−36^ dioxaborolane metathesis,^37−40^ and imine exchange.^41−46^ In addition to the various types of bond-exchange chemistries, further tunability of the bond exchange has been investigated. These include, e.g., steric or electronic effects with regard to the reactive groups,^47−50^ neighboring-group participation,^6,21,39,51^ or effects from the accompanying polymer matrix.^52−55^

An ongoing challenge for CANs is to strike a balance between allowing the material to be processed at elevated temperature (i.e., the processing temperature) and, while at the operating temperature (typically room temperature), keeping the material robust, displaying no creep.^56^ A variety of approaches has been explored to achieve this combination: e.g., by combining different types of dynamic covalent bonds (each with their own temperature-dependent exchange profile),^57−63^ adding nondynamic cross-links,^56,64^ or relying on a dynamic covalent bond motif with a dual-temperature response.^65^

Apart from these more chemistry-based approaches, recently more physical approaches relying on aggregation and phase separation also have been proposed.^66−73^ For linear polymers the effects of phase separation have already been thoroughly studied over several decades,^74−78^ and results have revealed an distinct enhancement in material properties: e.g., Matyjaszewski and co-workers reported on phase separation in poly(*tert*-butyl acrylate)-*block*-polystyrene (PBA-*b*-PS) copolymers^79^ and revealed that nonphase-separated materials showed a rapid decrease in storage modulus (*G*′) when heated and started flowing at ∼120 °C, while phase-separated materials exhibited an extended rubbery plateau with a *G*′ of ∼30 MPa and started to flow at ∼180 °C. In an analogous fashion, several promising approaches to induce phase separation into CANs for enhanced material properties have been developed. For example, grafting of dynamic covalent motifs onto incompatible polymer backbones has been found to result in phase separation.^80−82^ In addition, potential polarity effects and hydrogen bonding by the polymer backbones have been suggested as a cause for phase separation.^29,83,84^ Phase separation was also observed for block copolymers that incorporated coexisting dynamic and nondynamic blocks^54,85^ or in polymer networks with blended rigid and soft polymers.^86^ A general response of these phase-separated CANs was that the material properties of the bulk materials could be significantly enhanced, which can be expressed in, for example, a higher Young’s modulus^29^ or better creep resistance.^54^ As such, a better understanding of the process and effects of phase separation in CANs can be key to a new handle in the design of robust—yet dynamic—materials.

In the earlier examples the polymers were designed such that a phase separation was intentionally induced, for example, by selecting incompatible structures or by mixing amorphous and crystalline components. In contrast, the polyimine CANs reported herein were found to display phase separation despite being built up from miscible monomers, which arguably should yield homogeneous materials. We were able to visualize this phase separation of different dynamic domains in the range of several micrometers with Raman confocal spectroscopy. While regularly used for conventional polymers to study, for example, the polymer structure,^87^ polymerization kinetics,^88^ or crystallinity,^89^ the use of Raman spectroscopy in CANs has largely been overlooked, with the few reported examples of usage being limited to verification of formed bonds or structures.^90,91^ To the best of our knowledge, we are the first to apply Raman imaging to construct 2D images to reveal chemically different, phase-separated domains within the CAN bulk. We were able to construct these images owing to characteristic and unique bond energies of chemically distinctive imines and aromatic moieties. The fact that Raman could distinguish between very slight differences in molecular composition makes it a very powerful tool to construct images based solely on these small molecular differences (e.g., between different aromatic or imine signals).^92,93^ The method is also largely insensitive to the physical appearance of the material. For example, factors such as surface roughness or other inhomogeneities have little impact on the construction and quality of the Raman images. This chemically selective, yet robust, method is also what makes it different from other conventional techniques to study phase separation, such as atomic force microscopy (AFM), scanning electron microscopy (SEM), or X-ray diffraction (XRD). The further ease of the Raman setup and the relatively short time it takes to construct the Raman images (generally within several minutes, depending on size and resolution of the image), add to the value of choosing Raman over other methods.^94^ In addition, the minimal sample preparation for Raman spectroscopy is a major advantage.^93^ Samples as thin as a monolayer can be analyzed, whereas there is (technically) no upper limit in sample thickness. Raman scattering can, however, only be excited as far in the sample as the light can penetrate.^95^ The Raman imaging was able to provide detailed images on the micrometer scale, which is a typical length scale for this method and still above the diffraction limit of the resolution. Limitations occur below 100 nm. It should also be noted that, while Raman imaging proved especially useful in the analysis of the different aromatic and imine structures in this work, in other systems the overlap of distinctive signals might potentially cause problems to distinguish specific groups.

**Figure 1:**
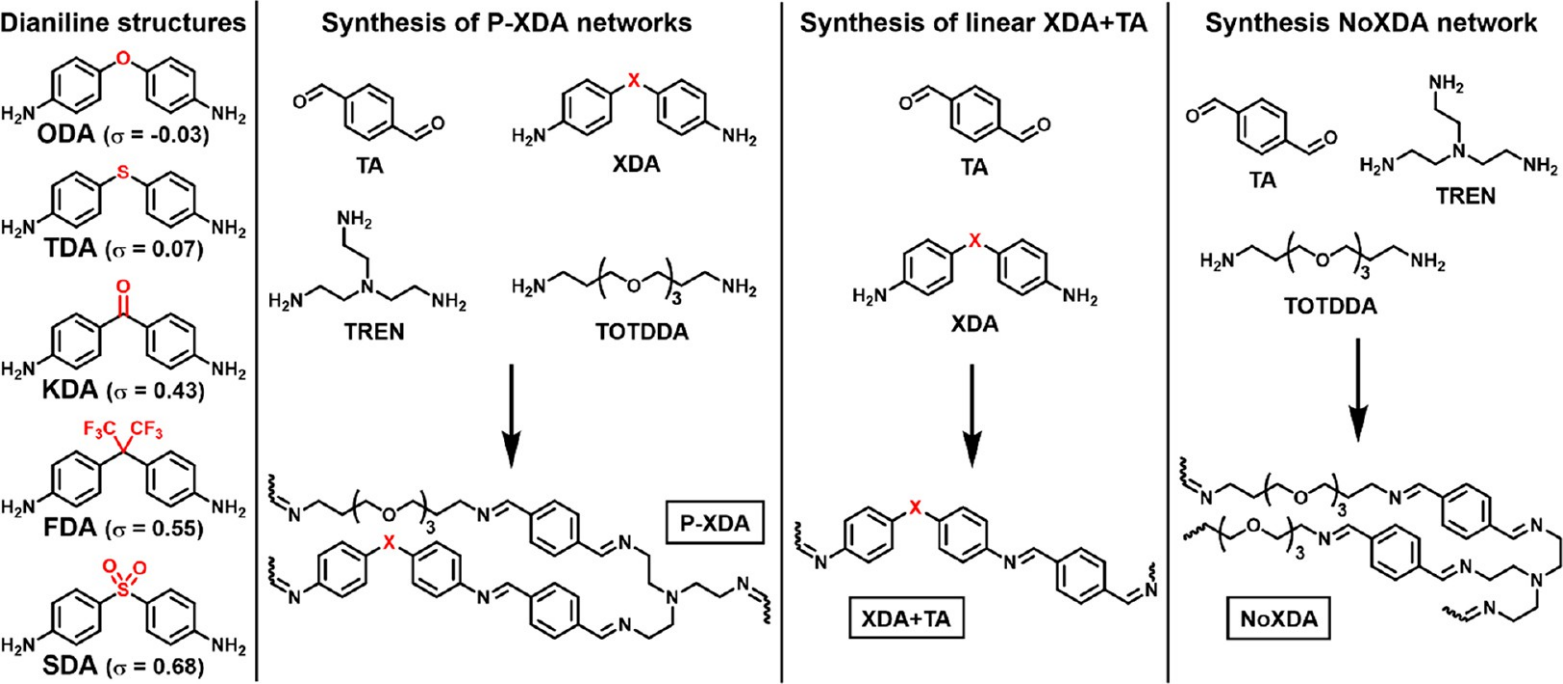
Overview of the building blocks for the synthesis of polyimines. The **P-XDA** polymer networks contain dialdehyde **TA**, cross-linker **TREN**, flexible linker **TOTDDA**, and either one of the five **XDA** dianilines shown on the left. Linear **XDA**+**TA** polymers do not contain **TOTDDA** or **TREN**. The **NoXDA** network does not contain any dianilines. The Hammett parameter (σ) of each **XDA** monomer expresses the electron-withdrawing effect of the bridging substituent (highlighted in red).^47^

The Raman imaging served as the key methodology for the visualization of phase separation in our polyimine CANs. First, we applied Raman imaging on earlier reported polyimines (Figure 1),^47^ revealing phase separation into different domains that were unknown to exist before. Second, by systematically exploring different compositions of the polymers, we found that the occurrence of the phase separation was directly related to the structure and concentration of the dianiline (**XDA**) monomer. The driving force for phase separation was found to be related to interactions between the aromatic components in the polymer network. Last, we also observed several noticeable changes in physical properties of the materials as a consequence of the phase separation. The phase separation generally resulted in mechanically more robust materials. First, the temperature at which the material transits from rubbery to viscous phase was greatly enhanced, and second, the elastic modulus of the materials was found to increase when phase separation was observed. We also observed that phase-separated materials appeared turbid, whereas nonphase-separated materials appeared transparent.

## Results and Discussion

### Raman Spectroscopy

For the analysis of polyimines with Raman spectroscopy, five different **P-XDA** polyimines were synthesized with dianiline contents of 20% following a previously published protocol^47^ (see Figure 1). The polymers were prepared by dissolving the amines (**XDA**, **TOTDDA**, and **TREN**) in a minimal amount of tetrahydrofuran (THF), to which the terephthalaldehyde (**TA**) then was added. The mixture was shaken briefly and then poured into a Petri dish. After solvent evaporation (first to air and later in a vacuum oven at 50 °C), the polymer films were obtained. These were subsequently analyzed by Raman spectroscopy (full experimental details are given in the Supporting Information).

**Figure 2:**
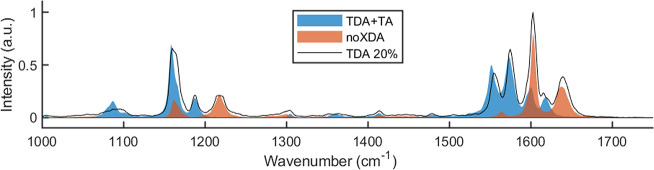
Overlapping Raman spectra of a linear polymer of **TDA**+**TA** (blue) and a polymer matrix of **TOTDDA**, **TREN**, and **TA** (**NoXDA**, orange). The combination of the two individual spectra matches that of the **P-TDA 20** material (black line).

In addition to the five **P-XDA** polymers from our earlier work,^47^ to elucidate the spectral features, several additional polymers were synthesized that contained selective parts of the full polymer composition. These additional reference polymers consisted of linear polymers of only dianiline and terephthalaldehyde (**XDA**+**TA**) and a polymer network with all components except the dianiline (**NoXDA**) (see Figure 1). The Raman spectra of all **P-XDA** materials were compared to the spectra of the corresponding **XDA**+**TA** and **NoXDA** materials (see Figure 2 for **P-TDA** and the Supporting Information for all other materials).

The Raman spectra showed three common regions in which Raman signals were observed: 0–200, 1100–1250, and 1500–1700 cm^–1^ (see the Supporting Information for full spectra of all **P-XDA** materials). The signals in the region of 0–200 cm^–1^ could be ascribed mostly to the aliphatic parts of the material and were found to be rather uninformative with respect to material composition. The other two regions contained the most characteristic data regarding the distinct aromatic moieties and imines. These include both common signals for all samples and sample-specific signals. Each individual signal of the **P-XDA** materials was then assigned to the corresponding bond vibration in the material based on data from the synthesized analogue materials (**XDA**+**TA** and **NoXDA**) and data of analogous structures reported in the literature.^96−101^ An overview of the assigned Raman signals is presented in Table 1, and a further elaboration is given in the Supporting Information.

**Table 1:** Assignment of Raman Signals to Corresponding Parts in the Polyimine Materials^a^

wavenumber (cm^–1^)	vibration	monomer(s)	reference(s)
1140–1160^b^	phenyl C–H bend	**XDA**	Badawi (2013),^98^ Nandi (2017),^99^ John (2018),^100^ Ullah (2019)^101^
1163^c^	phenyl C–H bend	**TA**	Nandi (2017),^99^ John (2018)^100^
1184–1190^b^	imine C–N stretch	**XDA**	Nandi (2017)^99^
1218^c^	imine C–N stretch	**TOTDDA**, **TREN**	Nandi (2017)^99^
1557–1560^b^	phenyl C=C stretch	**XDA**	John (2018)^100^
1563^b^	phenyl C=C stretch	**TA**	John (2018)^100^
1584–1585^b^	phenyl C=C stretch	**XDA**	Lee (2003),^96^ Ullah (2019)^101^
1602^c^	phenyl C=C stretch	**TA**	Lee (2003),^96^ Ullah (2019)^101^
1619–1624^b^	imine C=N stretch	**XDA**, **TA**	Nandi (2017),^99^ John (2018)^100^
1638^c^	imine C=N stretch	**TOTDDA**, **TREN**, **TA**	Lee (2003),^96^ Knöpke (2010),^97^ Nandi (2017),^99^ John (2018)^100^

a All signals were assigned by comparison to synthesized analogues of **TA+XDA** and **NoXDA** (Supporting Information) and to reported spectra of analogous materials.

b Indication that the signal is sample-specific.

c Indication that the signal is coherent for all materials.

### Raman Imaging of **P-TDA**

When scanning the surface of the **P-XDA** materials using the Raman confocal microscope, we recorded a 2D spectroscopic map and found two distinctly different spectra. These spectra resembled that of either the **XDA**+**TA** or the **NoXDA** material. This suggests two domains in the material, one containing mostly the aliphatic components (spectrum corresponding to **NoXDA**, in which only aliphatic imines, formed by reaction between **TOTDDA**, **TREN**, and **TA**, are present) and one containing mostly aromatic components (spectrum corresponding to **XDA**+**TA**, in which only aromatic imines, formed by reaction between **XDA** and **TA**, are present). To distinguish between the two types of spectra, the accompanying WITec software determined for every pixel to which of the two reference spectra the recorded spectrum was best fitted, and the pixel was colored accordingly. For all further analyses we assigned a yellow color to a best fit of **XDA**+**TA** and a blue color to a best fit of **NoXDA**. Also, we ruled out that no additional third distinct spectrum was observed that could point to other compositions (see the Supporting Information (Sections 4–6) for further elaboration on the procedure).

**Figure 3:**
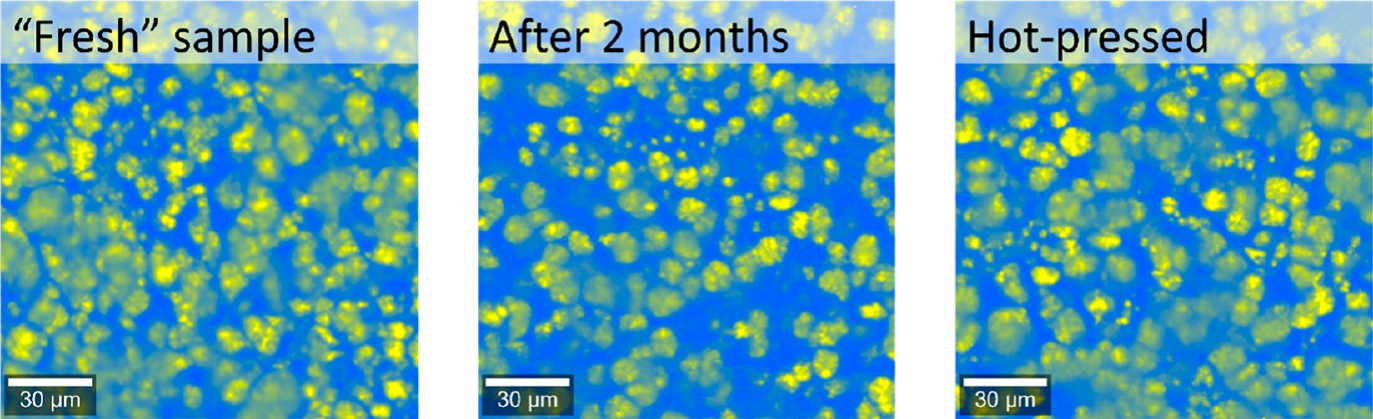
Raman images of **P-TDA 20** samples, directly after the synthesis (left), after 2 months (middle), and after hot-pressing (right). The yellow crystal-like domains indicate the regions where **TDA**+**TA** is predominantly present, and the blue regions represent a matrix of **TOTDDA**+**TREN**+**TA** (**NoXDA**) around the separated domains.

The first material for which such a Raman image was constructed was **P-TDA** (Figure 3, left). The image revealed a clear phase separation between yellow and blue domains, in which the yellow-colored domains (**TDA**+**TA** phase), which had a size of ∼10–20 μm, separated from the blue-colored matrix (**NoXDA** phase). To verify whether the phase separation remained stable over time, a new Raman image was constructed after 2 months (Figure 3, middle). This image showed a similar phase-separated profile and thus confirmed stability of the phase separation over time. Next, a sheet of **P-TDA** material was cut into smaller pieces and hot-pressed (100 °C for 30 min) into a new recycled polymer film. The Raman image of this recycled film also gave the same result (Figure 3, right). These results thus suggest that the phase separation is thermodynamically favorable as it persists over time and after thermal reprocessing.

**Figure 4:**
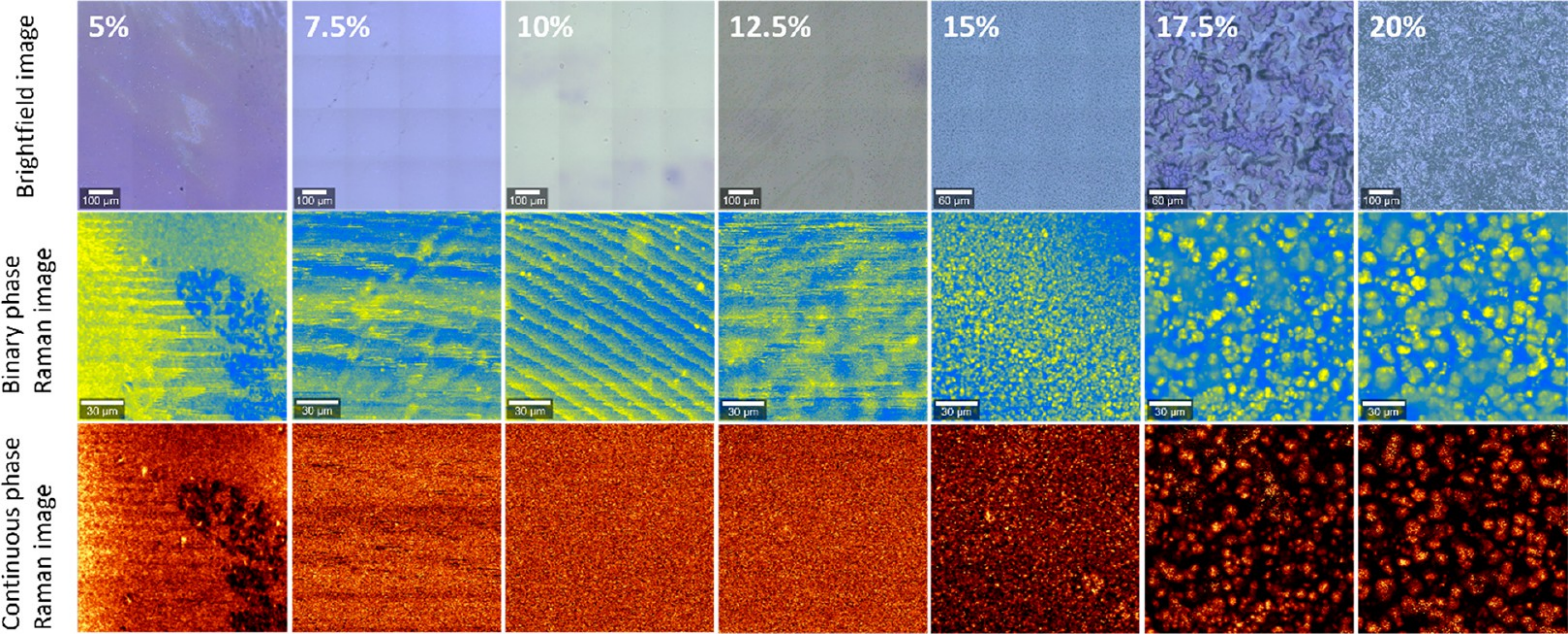
Brightfield and Raman images of **P-TDA** materials with varying dianiline concentrations from 5% to 20% (left to right, respectively). For the Raman images, a division was made between images constructed by applying either a binary fitting scale (middle row) or a continuous scale (bottom row).

To further investigate the occurrence of the phase separation, samples of **P-TDA** were prepared with lower concentrations of the dianiline monomer (i.e., lower relative amount of aromatic versus aliphatic segments). Both brightfield and Raman images were recorded for each of the samples (Figure 4). The brightfield images suggested a certain surface pattern for the **P-TDA** samples with 17.5% and 20% dianiline contents, but conclusive evidence for phase separation was only seen from the Raman images. These images showed that above 17.5% **TDA** phase separation could be clearly observed. At 15% the phase separation was still observed, although the domains appeared smaller in size. Below 12.5% **TDA** no clear phase separation was observed anymore, but rather a blurry image was obtained. These blurry images were the result of the forced assignment over two components. When the sample is homogeneous (no phase separation), this forced division into two different domains can show false patterns. This artifact can be seen in the 10% **TDA** sample. Although the initial binary fit can serve as a fast check to reveal phase separation, the assignment can be further supported by an extension of the analysis to a continuous-scale fitting procedure. Such a continuous scale can also give more information on the exact composition of the phases. In other words, a continuous scale to characterize the relevant features in the Raman spectra no longer provides an all-or-nothing determination of two phases but can show how strict the border is between the phases. To construct continuous-phase images, we introduced a 256-step scale based on peak area ratios in the Raman spectra, which were performed for each individual pixel (see the Supporting Information for additional experimental details). The continuous-scale Raman images could generally nicely filter noise and artifacts (especially when there was no phase separation), although the choice for a binary fit could in most cases more clearly (and quickly) show the contrast between phases when phase separation was observed. For the best representation of results, we therefore generally included both binary and continuous-scale Raman images.

From the processed Raman images, we could then conclude that phase separation did not occur for any of the samples with a **TDA** concentration of 12.5% and below. At 15% **TDA** we observed the first hints of phase separation, which became clearer when further increasing the concentration of **TDA**. These results thus suggest the existence of a critical concentration of 15% **TDA**, above which the phase separation occurs. Below this threshold concentration the dianiline parts can still blend in with the rest of the matrix; above the threshold they separate into isolated domains. Despite the crystal-like appearance of the phase-separated **TDA**-rich domains, DSCs of 10% and 20% **TDA** materials did not reveal any thermal transition that could be assigned to the melting transition of the phase-separated domains or the accompanying matrix (see the Supporting Information).

**Figure 5:**
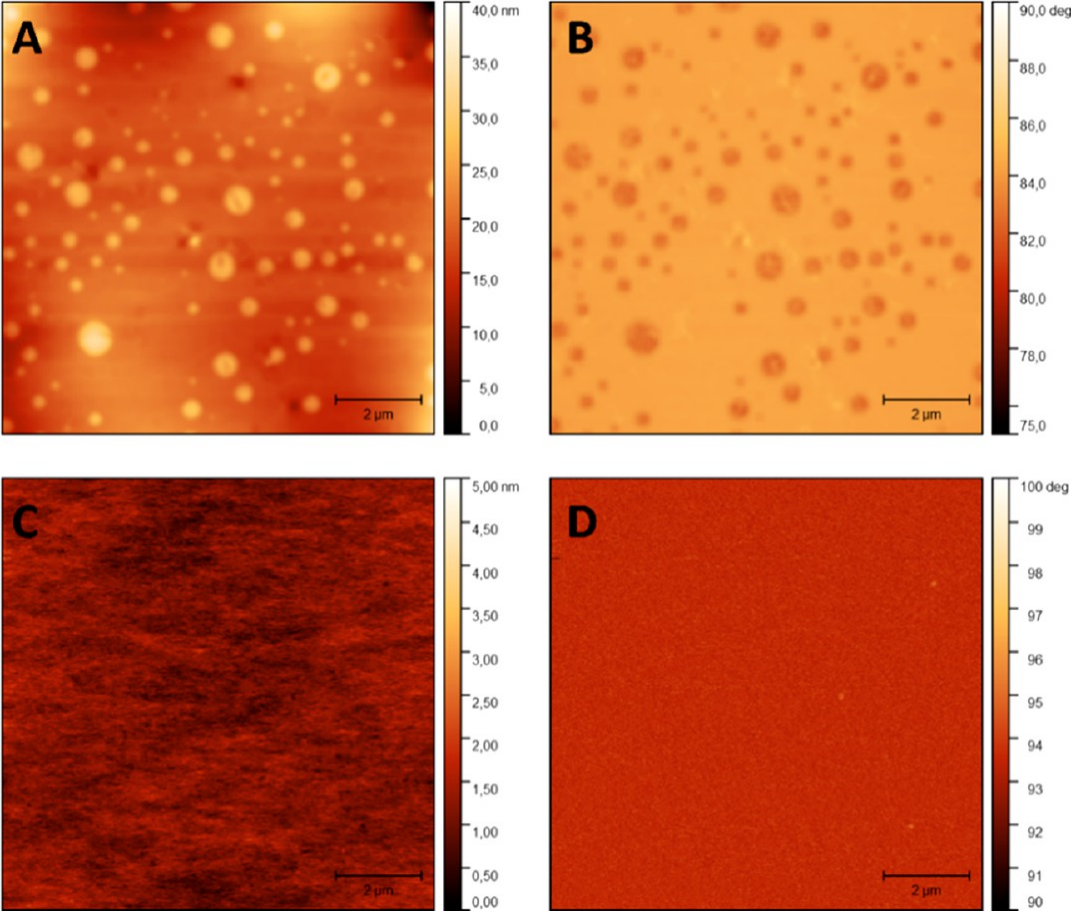
AFM images of **P-TDA** materials: (A) height profile of **P-TDA 20**, (B) phase profile of **P-TDA 20**, (C) height profile of **P-TDA 5**, and (D) phase profile of **P-TDA 5**. The AFM images show a clear phase separation for **P***-***TDA 20**, whereas for **P-TDA 5** no phase separation was observed, confirming the results from the Raman images. Note the different height scales in panels A and C.

As an additional analytical method to confirm phase separation, atomic force microscopy (AFM) was applied. First, height (Figure 5A) and phase (Figure 5B) images of **P-TDA 20** were constructed. The AFM images showed a similar phase separation in sphere-like hard domains entrapped in a soft matrix. The size of the phase-separated domains appeared smaller than was observed with Raman spectra, although this is likely the result of the AFM only measuring at the surface of the film and, as such, only observing the top of the spherical hard phases. In contrast, with Raman spectroscopy, the entire upper layer of the film (up to several micrometers) can be visualized. Next, AFM images were constructed of **P-TDA 5** (parts C and D of Figure 5 for height and phase, respectively), which revealed the absence of phase-separated domains at low **TDA** concentrations.

**Figure 6:**
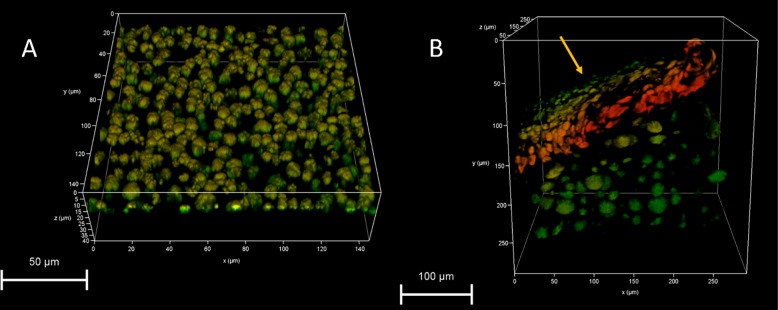
(A) 3D multiphoton fluorescence lifetime image of the surface of **P-TDA 20**, and (B) cross-sectional image of the **P-TDA 20** sample (the yellow arrow indicates the surface of the sample). The colored parts show the phase-separated domains of **TDA**+**TA**; the red color indicates nearby phases, and the greener colors indicate phases further away.

To further study the sizes and shapes of the phase-separated domains, Raman scans of **P-TDA 15** and **P-TDA 20** were constructed slightly deeper below the surface up until roughly 10 μm (see the Supporting Information). In these images, the phase-separated domains gradually increase in size by several μm (ultimately doubling in size) when moving deeper into the sample. To study the dimensions and distribution of the domains in greater detail, 3D multiphoton fluorescence lifetime microscopy was used. A 3D image of **P-TDA 20** was constructed by recording the fluorescence that specifically arose from the distinctive aromatic moieties in the system (Figure 6). The image shows phase-separated domains with an approximately spherical shape on a similar scale as was observed by Raman. A cross-sectional image (Figure 6B) indicated that the phase-separated domains appeared smaller and more densely packed near the surface of the film compared to the phase-separated domains deeper in the sample, in line with the observation of smaller domains by AFM (Figure 5). This suggests different dynamic behavior between the CAN surface and bulk, as has been proposed in recent work by Liu and Wang.^102^ The multiphoton images thus first served as additional experimental evidence for the observed phase separation previously observed with Raman imaging and furthermore supplemented new information regarding the size and distribution of phase-separated domains deeper in the material bulk.

We considered that solvation and drying effects during the formation of the polymer films might affect the phase-separation process.^103−106^ In an attempt to study the formation of polymer clusters in solution, dynamic light scattering (DLS) was applied. Solutions were prepared by mixing the monomers for several phase-separating and nonphase-separating materials, and with DLS we checked for the formation and size of formed particles in solution over time (see the Supporting Information for experimental details). Unfortunately, these results could not reveal any underlying dynamics regarding the formation of particles that could be linked to phase separation.

Next, in addition to the standard preparation of the polymer films in THF,^47^**P-TDA 20** films were prepared in different solvents: 2-methyltetrahydrofuran (2-MeTHF), methanol (MeOH), ethyl acetate (EtOAc), or toluene. Brightfield and Raman images were constructed of the polymer films (see the Supporting Information), which all showed a similar phase separation as was seen before. We also prepared several thinner films of **P-TDA 20** to check for potential effects of film thickness on the size and distribution of the microdomains (see the Supporting Information) but could not identify any observable differences either. The consistency of the combined results underlines the robustness of the preparation for the polyimine materials, as it appears that neither the scale nor the solvent in the synthesis seemed to affect the occurrence of the phase separation. In favor of this result, in future work we could thus easily adjust the scale of the preparation or switch to greener solvents, such as 2-MeTHF, without observable negative consequences for the prepared polymer material.

### Phase Separation for Other Dianilines

**Figure 7:**
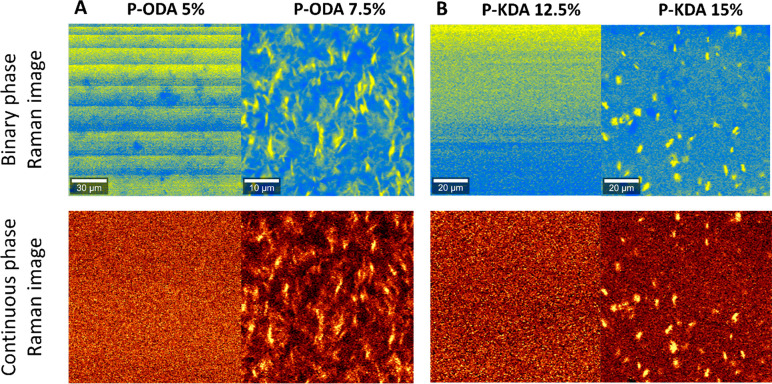
Raman images of (A) **P-ODA** and (B) **P-KDA** samples. The images show that for **P***-***ODA** phase separation occurs above a dianiline content of 7.5%, while for **P-KDA** a dianiline content of 15% is required at minimum. Binary and continuous color coding of the phases are included.

After studying the phase separation for the **TDA**-based polymers, the other four dianilines from our earlier work^47^ were used to prepare their corresponding CANs (see Figure 1), and Raman images were again recorded. First, **ODA** was investigated as this was structurally the most similar dianiline to **TDA**. The Raman images of **P-ODA** revealed a clear phase separation, although some differences compared to **P-TDA** were noted. First, the phase separation occurred already at a dianiline concentration of 7.5% for **P-ODA** (Figure 7A), compared to 15% for **P-TDA** (Figure 4). Second, the shape of the phase-separated domains was slightly different: for **P-ODA** they appeared needle-like, whereas for **P-TDA** they appeared sphere-like. In terms of their chemical structure, **TDA** and **ODA** are rather similar (Figure 1). However, the Hammett parameter (σ) for **ODA** is higher than that of **TDA**. This translates to a higher electron-donating effect from the **ODA** toward the imines, which in turn affects the kinetics of the bond exchange.^47^ From this perspective, the internal kinetics of the bond exchange might play a role in the character of the phase separation.

Next, the more electron-withdrawing **KDA** monomer was investigated. The Raman images of **P-KDA** indicated a similar phase separation to **P-TDA** with the threshold for the phase separation at 15% dianiline content (Figure 7B), although the phase-separated domains appeared slightly smaller in size. Surprisingly, materials of the two most electron-withdrawing dianilines (**FDA** and **SDA**) did not show any observable phase separation, even when increasing the dianiline content to as high as 50% (see the Supporting Information). We considered two possible hypotheses for these observations. First, because of the strong electron-withdrawing nature of the substituents, the stability of the imines is decreased in the cases of **FDA** and **SDA**,^107,108^ and as such the susceptibility toward exchange reactions increases.^47^ This increased reactivity results in more efficient bond exchange with the imines from the aliphatic matrix^109^ and, as such, could prevent separation into different phases. Alternatively, the steric bulk of the **FDA** and **SDA** monomers is (slightly) higher compared to the other three (**ODA**, **TDA**, and **KDA**) monomers. This increased steric bulk of the variable moiety in the **XDA** monomer may prevent efficient stacking of the two aromatic rings of the **XDA** monomer and therefore hamper the phase-separation process.

**Figure 8:**
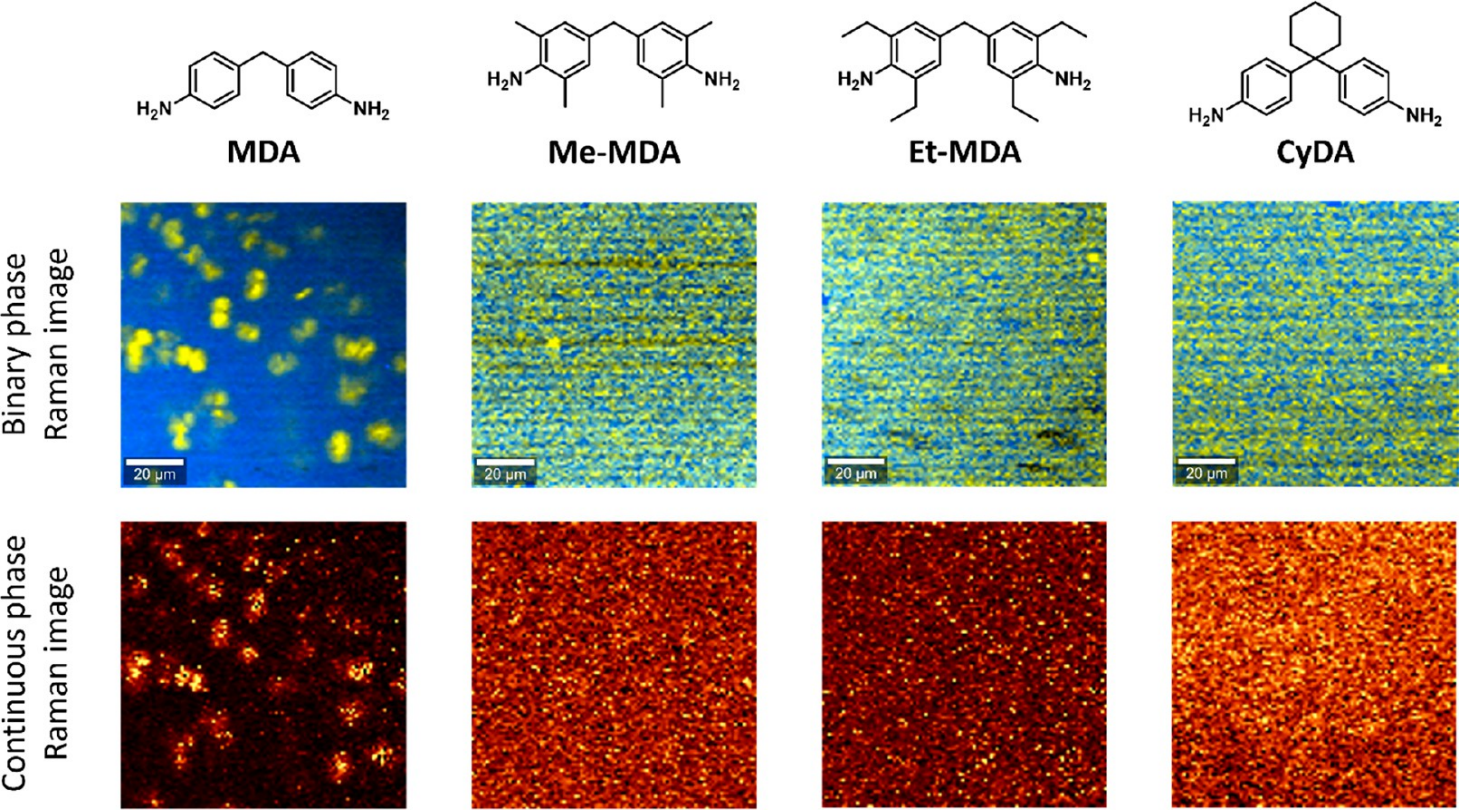
Structure and Raman images of dianilines with different substituents varying in steric hindrance. The Raman images show that phase separation was clearly observed for the nonhindered **P-MDA** but not for the sterically hindered analogous materials. For all materials the dianiline content was 20%. Binary and continuous color coding of the phases are included.

To further study the possible steric effects of the dianilines on the phase separation, several other (commercially available) dianilines were selected to synthesize additional materials (Figure 8). First, a sterically nonhindered 4,4′-methylenedianiline (**MDA**) was selected to serve as a reference. If the phase separation would be caused by electronic effects, based on the Hammett parameter for this monomer,^110−112^ it is expected to show a similar phase separation as **P-TDA** and **P-ODA**. This was also confirmed from the Raman image (Figure 8). Then, several additional polymer films were prepared from dianilines with a similar electronic effect but increased steric bulk. To this end, we first selected dianilines with alkyl groups (methyl or ethyl for **Me-MDA** and **Et-MDA**, respectively) on both ortho positions next to the amine group on the aromatic ring. In addition, we selected a dianiline monomer with a bulky cyclohexyl substituent on the bridging sp^3^ carbon (**CyDA**). Raman images from these materials concluded that none of them showed phase separation, strongly suggesting that steric effects in relation to π–π stacking indeed affect the occurrence of phase separation. These observations are also in line with results from ureidopyrimidone (UPy)-based supramolecular polymers, for which a disappearance in microphase separation was noticed with increasing steric hindrance on the pyrimidone moieties.^113,114^ It was demonstrated that the branching effect of the substituents suppresses aggregation, as larger steric groups hinder efficient π–π stacking and prevent phase separation. Similarly, the branched dianilines in our study could suppress aggregation of **XDA**+**TA** domains and prevent phase separation.

**Figure 9:**
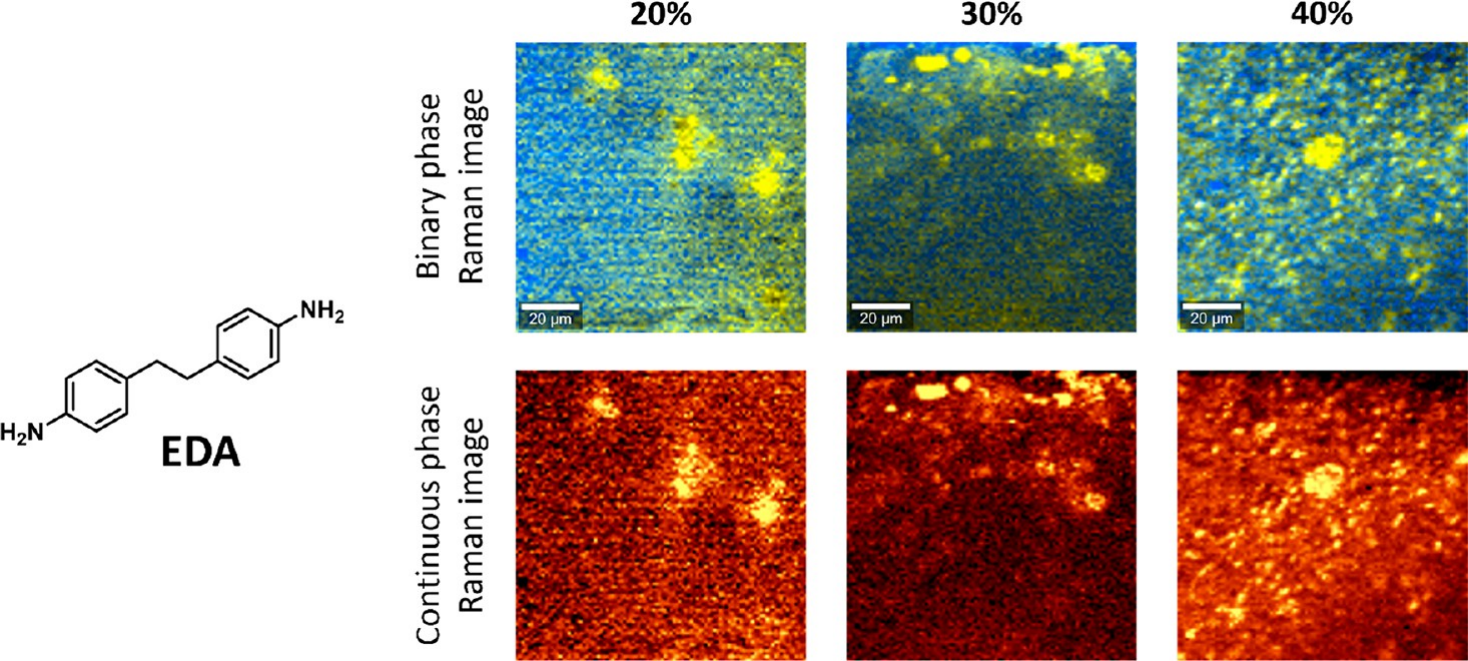
Raman images of **P-EDA** materials with 20, 30, and 40% dianiline content. Phase separation was observed for all samples, although less clearly than was seen for **P-MDA**. Binary and continuous color coding of the phases are included.

A final dianiline monomer was selected that contained a longer ethylene moiety as a bridge (**EDA**) (Figure 9). The Hammett parameters for methyl (σ = −0.17) and ethyl (σ = −0.15) substituents are similar;^110^ however, the rotational freedom of the ethylene is higher due to the longer chain length. As a result, the increased flexibility could affect the stacking of the aromatic moieties. The Raman image of **P-EDA** did indicate phase separation, although less clearly than was seen for **P-MDA**. Phase-separated domains were observed, but the matrix around them did not appear to be fully free of the **EDA** signal, which suggests mixing of **EDA** with the rest of the matrix. Increasing the dianiline content to 30% and 40% yielded similar Raman images. The lesser degree of phase separation for **P-EDA** compared to **P-MDA** may be explained by the difference in the rigidity of the two monomers. While these two dianilines are more rigid than the other aliphatic amines in the polymer matrix, the phase-separation process leads to a division in “hard” aromatic segments versus “soft” aliphatic segments.^115,116^ However, when switching from **MDA** to **EDA**, the dianiline part becomes less rigid due to the added length and flexibility of the monomer, which results in a smaller driving force for phase separation. To summarize, these studies indicate that the phase separation is—at least in part—driven by aromatic interactions between the dianiline (**XDA**) monomers and can be disrupted by steric effects that hamper these aromatic interactions.

### Material Consequences of Phase Separation

**Figure 10:**
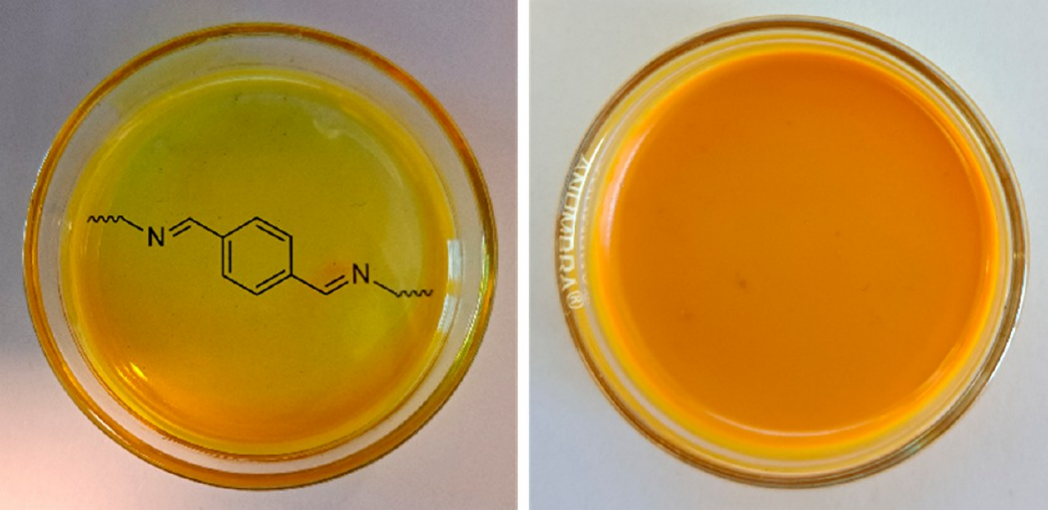
Difference in transparency between **P-TDA** samples. On the left a transparent sample (**P-TDA 10**), and on the right a turbid sample (**P-TDA 20**). All samples with a dianiline content of 15% **TDA** or higher appeared turbid, and samples with a dianiline content of 12.5% **TDA** or lower appeared transparent (see the Supporting Information for all materials). Pictures were made directly after synthesis (including drying), with the samples still in the Petri dish in which they were synthesized. The thickness of the films is ∼0.5 mm.

Having observed the phase separation in our polyimine materials, we investigated its effects on the polymer’s material properties. For this we first made use of our set of **P-TDA** materials with dianiline contents from 5 to 20%. First, a simple visual inspection of the samples revealed that they all had a bright yellow-to-orange color that is characteristic to (poly)imines.^47,52,117,118^ However, the samples above the threshold dianiline concentration (>15% **TDA**) for phase separation appeared turbid, whereas the samples below the threshold (<12.5% **TDA**) appeared transparent (Figure 10). Looking at the samples from the **P-MDA** and related sterically bulked materials, a similar observation was made in which **P-MDA** and **P-EDA** appeared turbid, while the sterically hindered analogous materials (**P-MeMDA**, **P-EtMDA**, and **P-CyDA**) were transparent (see the Supporting Information for photos of the materials). The transparency of the materials gives a good initial indication of the microstructure of the polymer network as transparency is commonly linked to amorphous structures.^72^ It is also likely that turbid samples contain (semi)crystalline scattering domains in their network structure on the order of the wavelength of visible light.

All 5–20% **P-TDA** samples were then individually analyzed with a rheology setup by performing a temperature-sweep experiment and a creep test (see the Supporting Information for full experimental details). From the temperature sweeps the crossover point between *G*′ and *G*′′ (i.e., where tan(δ) = 1) was determined, representing the temperature above which the material transitions from a viscoelastic solid to a viscoelastic liquid (i.e., the material starts to flow).^119,120^ For CANs, the description of this point is not as trivial as for traditional thermoplastics. This is because (associative) CANs do not fully melt, as the cross-linking density remains constant at elevated temperatures.^47^ Instead, the malleability of the material is a function of the reaction rate of the bond exchange.^121^ When at a certain point the viscous component (*G*′′) becomes more prominent than the elastic modulus (*G*′), this simply translates to the material showing a higher tendency to permanent deformation as a result of the stress–relaxation via bond exchange rather than to an elastic response, when a strain is applied. In this work, we will refer to this point where *G*′′ exceeds *G*′ (i.e., tan(δ) = 1) as the crossover temperature (*T*_cross_).^120^

**Figure 11:**
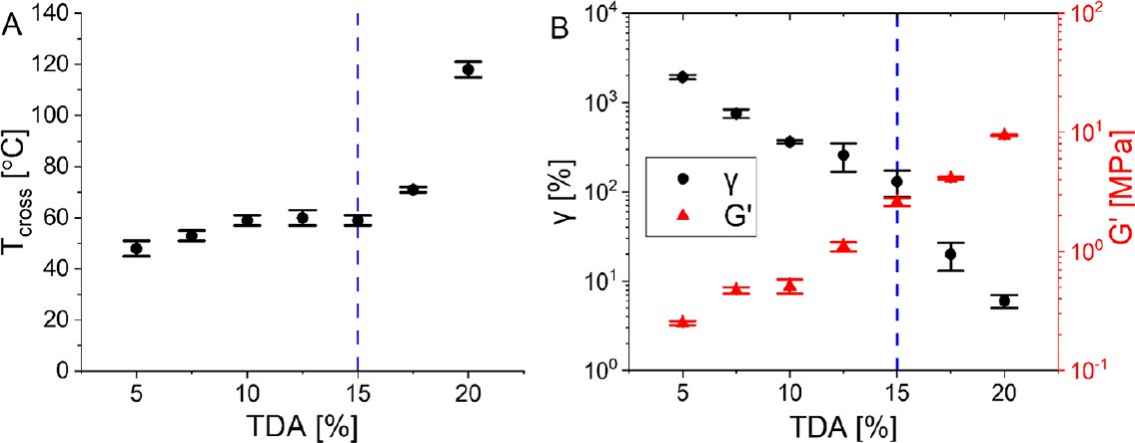
(A) Plot of *T*_cross_ as a function of the **TDA** content from 5 to 20%. (B) Plot of the creep after 1000 s at a 10 kPa stress (black spheres) and elastic modulus (*G*′, red triangles) at room temperature, as a function of the **TDA** concentration. The dotted blue line indicates the threshold value for phase separation at 15% **TDA**.

Around the threshold for phase separation (here from 10–15% **TDA**), the *T*_cross_ remains constant at ∼60 °C (Figure 11). Above 15% the *T*_cross_ quickly rises to much higher temperatures, while below 10% the *T*_cross_ slightly drops. Especially, the steep rise in *T*_cross_ after overcoming the threshold for phase separation gives a clear indication of the effect of phase separation on the material properties. The most likely explanation for this strong increase in *T*_cross_ is that the phase separation causes the formation of local hard domains with increased rigidity that could hamper strand diffusion and overall network deformation,^54^ leading to an increased apparent (noncovalent) cross-linking density.^122^ Potentially, hydrophobic interactions and π–π stacking of the aromatic moieties in the phase-separated domains increases the required energy to mobilize the polymer chains.^123^ As such, a higher temperature is needed to induce mobility, which is expressed in the increased *T*_cross_. Furthermore, the reduced chain mobility hinders the occurrence of bond-exchange reactions, leading to reduced dynamicity and chain rearrangements.^52,124^

Other commonly reported results of phase separation in polymers are enhanced creep resistance and dynamic moduli.^85,125^ The results of the creep tests of the **P-TDA** samples (Figure 11B, black data points) showed a significant resistance to creep when the **TDA** concentration increased, although it was difficult to identify a clear transition at the onset of phase separation. An increase in **TDA** content also means that the relative concentration of **TOTDDA** decreases, which results in a higher cross-linking density and lower overall strand flexibility. This implies that the resistance to creep is expected to increase upon higher **TDA** concentrations. The same applies to the elastic modulus (*G*′) of the materials (Figure 11B, red data points). One might argue that the elastic modulus rises faster after the threshold for phase separation, although the thus far obtained data were not yet sufficient to substantiate if the observed changes were selectively caused by phase separation or if the small differences in ratio between the monomers could have an effect here as well. To further claim the direct effect of the phase separation and rule out other effects such as the concentration of the respective monomers, additional experiments were performed in which we compared the differently substituted **P-MDA** materials (see Figure 8).

**Figure 12:**
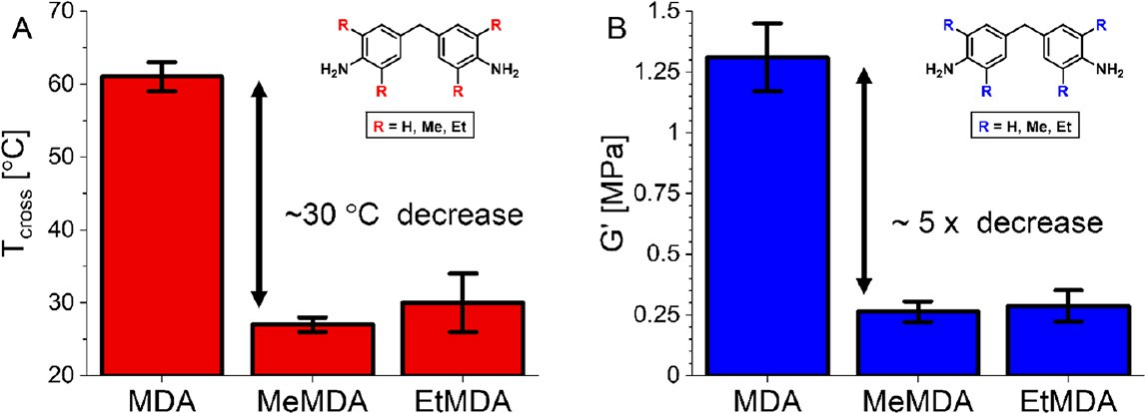
(A) *T*_cross_ values for phase-separated **P-MDA** and sterically hindered nonphase-separated **P***-***MeMDA** and **P-EtMDA**. It can be seen that the *T*_cross_ drops by as much as ∼30 °C when the phase-separated profile is lost. (B) *G*′ values for phase-separated **P-MDA** and sterically hindered nonphase-separated **P***-***MeMDA** and **P-EtMDA**. It can be seen that the *G*′ decreases by a factor ∼5 when the phase-separated profile is lost.

The same rheological tests as before were performed on **P-MDA**, **P-MeMDA**, and **P-EtMDA** materials with a constant composition of 20% of the respective dianiline. By using this set of materials, we could rule out the effects related to the difference in aromatic/aliphatic content as was seen for the **P-TDA** materials with different dianiline contents. From temperature-sweep experiments a similar effect on the *T*_cross_ was first noticed. For the nonsterically hindered and phase-separated **P-MDA**, a *T*_cross_ of 61 ± 2 °C was found, but for the sterically hindered and nonphase-separated **P-MeMDA** and **P-EtMDA**, this value markedly dropped to roughly 30 °C (Figure 12A). This time, a noticeable effect of the phase separation on the elastic modulus (*G*′) also was observed. The nonphase-separated **P-MeMDA** and **P-EtMDA** had similar *G*′ values of 0.26 ± 0.04 MPa and 0.29 ± 0.06 MPa, respectively. However, the phase-separated **P-MDA** showed a roughly 5-fold increase in *G*′ to 1.31 ± 0.14 MPa (Figure 12B). This increase in *G*′ is most likely a direct consequence of the phase separation because the concentrations of the aromatic dianilines in all materials were the same, and as such the average cross-linking density and chain rigidity in the material can be considered identical.

By combining all of these results, we can thus claim that the phase separation had several consequences to the material properties of the polyimine CANs. First, the transparency is affected as phase-separated materials become turbid, whereas nonphase-separated materials are transparent. Second, and more relevantly, the dynamic-mechanical properties of the materials are affected, as a significantly higher temperature is required to transition the materials from a rubbery to a viscous state (expressed in *T*_cross_) once phase separation occurred. Also, the elastic moduli of the materials increased markedly for the phase-separated materials with similar compositions with respect to the aliphatic/aromatic content.

### Reversing the Phase Separation

**Figure 13:**
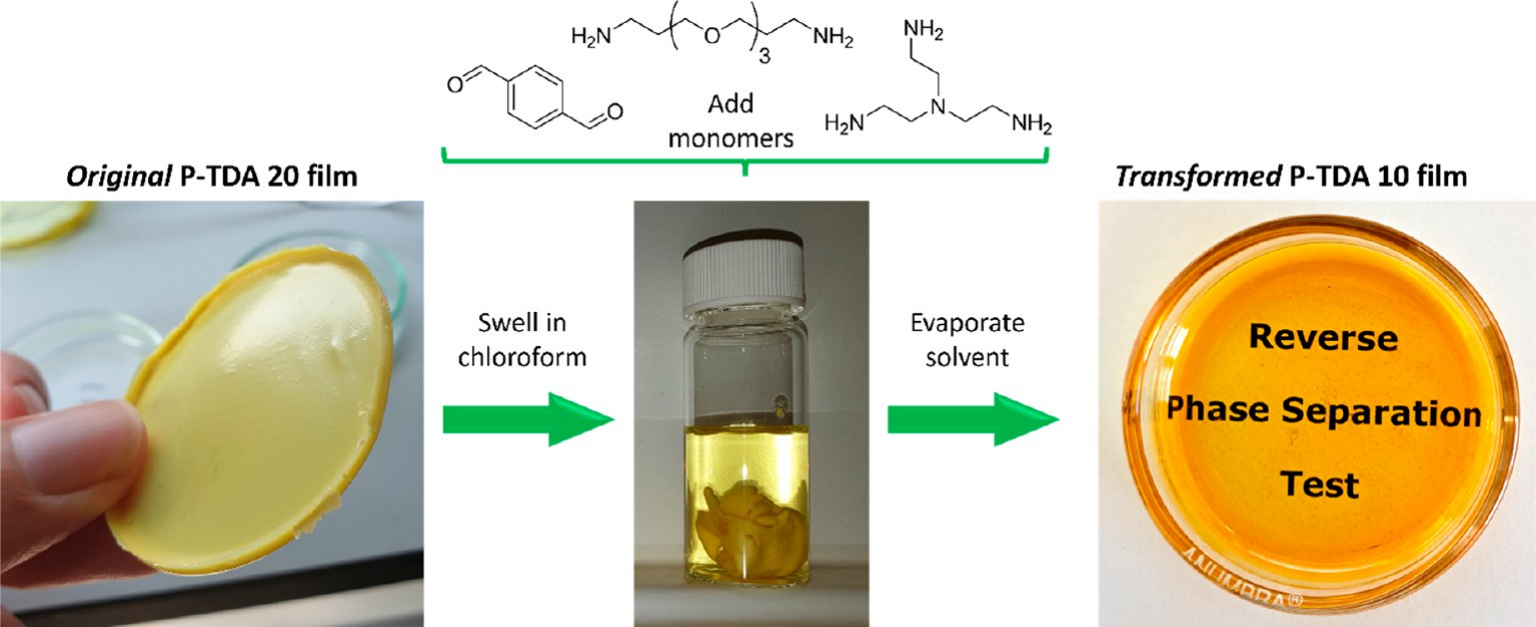
Reverse-phase-separation experiment to transform phase-separated **P-TDA 20** (left) into nonphase-separated **P-TDA 10** (right) by the addition of fresh monomers (**TA**, **TREN**, and **TOTDDA**).

As we concluded before, the phase separation of the **P-XDA** materials was dependent on the concentration of the **XDA** dianiline inside the covalent adaptable network. Relying on the intrinsically dynamic nature of this network, we envisioned that we could reverse the phase separation of phase-separated polyimines by addition of the other monomers and thereby decrease the overall **XDA** dianiline content. For this experiment we started with a phase-separated **P-TDA 20** film, with the goal to dilute this material to a nonphase-separated **P-TDA 10** film. To do so, we precisely weighed a piece of **P-TDA 20** material. We then added the appropriate amount of **TA**, **TOTDDA**, and **TREN** monomers in order to decrease the total **TDA** content from 20% to 10% (see the Supporting Information for calculations and precise amounts). The **P-TDA** film was then swollen in chloroform. To this sample, first a solution of **TOTDDA** and **TREN** in chloroform was added, and the mixture was shaken thoroughly. Then a solution of **TA** in chloroform was added, and the mixture was shaken again. The mixture was left overnight before the content of the vial was poured into a Petri dish. The Petri dish was left open to air for 24 h so that most of the solvent was able to evaporate to air, and a new polymer film was obtained. The newly obtained film was further dried in a vacuum oven at 50 °C for another 24 h. Then, the new transformed **P-TDA 10** film was analyzed. We found that the phase separation had indeed disappeared as Raman imaging no longer indicated phase-separated domains and the polymer was transformed from a turbid to a transparent film (Figure 13). This shows that the phase separation is a dynamic and reversible process, which is essential for the development and analysis of future CANs. It also further shows that the phase separation is a thermodynamically driven process that can be controlled and reversed.

## Conclusion

In this work we introduced Raman confocal spectroscopy to construct 2D images of polyimine CANs based on their chemical composition, leading to the discovery of a phase separation of mainly aromatic versus aliphatic domains. The occurrence of this phase separation was dependent on the chemical nature of dianiline monomers and only occurred above a threshold concentration of the dianilines. The phase separation could be reversed by employing the intrinsic dynamic nature of the imine network after phase separation, by reducing the dianiline concentration via swelling in the solvent and the addition of other monomers. Without phase separation the polyimine materials appeared transparent, while phase-separated materials appeared turbid. Also, a significant increase in *T*_cross_ was observed once the threshold concentration of dianilines for phase separation was crossed. Sterically demanding groups could be introduced on the dianiline monomer structure to prevent phase separation, and loss of the phase-separated profile caused both the *T*_cross_ and *G*′ to noticeably decrease. Overall, in line with the strengthening effect of phase separation, we demonstrate that this physical-chemical strategy can be used (and visualized by Raman imaging) to toughen polyimine CANs.

Knowledge of the occurrence and effects of phase separation is of major importance when designing dynamic polymer materials such as polyimines. We foresee that further work on the origin of phase separation in dynamic polymer materials could offer a new handle for physical-chemical tunability of material properties via a bottom-up approach, moving away from more chemical approaches operating at the nanometer scale to the micrometer scale at which phase separation occurs. Furthermore, our work shows that Raman imaging can be applied as a powerful tool to study phase separation in CANs. As such, we would like to stimulate its further application in the field of dynamic polymer materials.

## Supporting Information Available

Experimental details of synthesis and analysis, additional spectra and images (such as Raman spectra and images, brightfield images, DSC curves, DLS plots, rheology curves, and photos of materials), and more extensive visualization and elaborations of results (PDF)
