# Supplementary material for: Raman Spectroscopy Reveals Phase Separation in Imine-Based Covalent Adaptable Networks
Source: Macromolecules. 2022 Nov 30;55(23):10341–55. doi: 10.1021/acs.macromol.2c01595 (PMC9753755; doi:10.1021/acs.macromol.2c01595)
Supplement: Supplementary file 1 — ma2c01595_si_001.pdf [file ma2c01595_si_001.pdf]

Electronic Supporting Information for:

Raman Spectroscopy Reveals Phase Separation in Imine-based Covalent Adaptable Networks

Sybren K. Schoustra,[a] Martijn H. P. de Heer Kloots,[a,b] Joris Posthuma,[a,b] Daphne van Doorn,[a,b] Joshua A. Dijksman,[b,] Maarten M. J. Smulders[a,*]*

[a] Laboratory of Organic Chemistry, Wageningen University, Stippeneng 4, 6708 WE Wageningen, The Netherlands. E-mail: maarten.smulders@wur.nl

[b] Department of Physical Chemistry and Soft Matter, Wageningen University, Stippeneng 4, 6708 WE Wageningen, The Netherlands. E-mail: joshua.dijksman@wur.nl

* joshua.dijksman@wur.nl; maarten.smulders@wur.nl

Table of contents

Table of contents S2

1. Synthesis of polyimine networks S4

2. Experimental setup S6

2.1 Raman Spectroscopy S6

2.2 Rheology S6

2.3 Atomic force microscopy S7

2.4 3D multiphoton fluorescence lifetime microscopy S7

3. Assignment of Raman signals S9

3.1 Raman spectra of 20% P-XDA samples...S9

3.2 Raman spectra of **NoXDA** and **XDA+TA** analogue samples S10

3.3 Common signals from 1100–1250 cm^{-1} S11

3.4 Common signals from 1500–1700 cm^{-1} S11

3.5 Sample specific signals from 1100–1250 cm^{-1} S12

3.6 Sample specific signals from 1500–1700 cm^{-1} S12

4. Raman image analysis for fitting with a binary scale S13

5. Continuous coloring scale of Raman images S15

5.1 Procedure for creation of continuous-scale Raman images S16

6. Fitting without reference spectra and ruling out a third phase S18

7. Depth scans of 15 and 20% P-TDA samples S20

8. Brightfield and Raman images of P-TDA20 films with different thickness S21

9. Raman images of P-TDA20 samples, synthesized from different solvents S22

10. Dynamic Light Scattering (DLS) S23

11. Material properties of P-TDA samples with dianiline contents from 5 to 20%.....S26

12. Photos of P-XDA sample with regards to transparency..................................S27

13. Temperature sweep curves of P-TDA 5-20...S28

14. Temperature sweeps of P-MDA, P-MeMDA and P-EtMDA S30

15. DSC curves of P-TDA 10 and P-TDA 20 ....S31

16. Brightfield and Raman images S32

16.1 **P-ODA** samples S32
16.2 **P-KDA** samples S32
16.3 **P-FDA** samples S33
16.4 **P-SDA** samples S34
16.5 **P-EDA** samples S35
17. Reversing phase separation S36
18. References S37

1. Synthesis of polyimine networks

All polyimine materials were prepared according to previously reported synthetic procedure for polyimine CANs in our group:[S1] **TOTDDA**, **TREN**, and the corresponding dianiline (**XDA**) were fully dissolved in THF. Then **TA** was added, and the mixture was shaken thoroughly until all material was fully dissolved. The solution was poured into a petri dish, and the samples were left for overnight in the fume hood to evaporate most of the solvent. Afterwards the samples were further dried in a vacuum oven at 50 °C for at least 2 more days. See Tables S1–2 for full details of the used materials.

Table S1. Overview of used chemicals, abbreviations, purity and suppliers.

Material	Abbreviation	Purity	Supplier
4,4'-Oxydianiline	ODA	98%	Tokyo Chemical Industry
4,4'-Thiodianiline	TDA	98%	Tokyo Chemical Industry
4,4'-Diaminobenzophenone	KDA	97%	Sigma-Aldrich (Merck)
4,4'-(Hexafluoroisopropylidene)dianiline	FDA	98%	Acros Organics
4,4'-Diaminophenylsulfone	SDA	97%	Acros Organics
4,4'-Methylenedianiline	MDA	97%	Sigma-Aldrich (Merck)
1,1-Bis(4-aminophenyl)cyclohexane	CyDA	98%	Tokyo Chemical Industry
4,4'-Methylenebis(2,6-dimethylaniline)	Me-MDA	99%	Sigma-Aldrich (Merck)
4,4'-Methylenebis(2,6-diethylaniline)	Et-MDA	98%	Tokyo Chemical Industry
4,4'-Ethylenedianiline	EDA	97%	Sigma-Aldrich (Merck)
Tris(2-aminoethyl)amine	TREN	98%	Tokyo Chemical Industry
4,7,10-Trioxa-1,13-tridecanediamine	TOTDDA	97%	Sigma-Aldrich (Merck)
Terephthalaldehyde	TA	99%	Sigma-Aldrich (Merck)
Tetrahydrofuran	THF	>99%	Biosolve
Toluene	tol	>99%	Biosolve
Methanol	MeOH	>99%	Biosolve
Ethyl acetate	EtOAc	>99%	Biosolve
2-Methyltetrahydrofuran	2-MeTHF	>99%	Fisher Scientific

Table S2. Required monomer amounts for each polyimine material. All samples were prepared in 5 mL THF and mixed at room temperature.

**Material**	**XDA** (mmol)	**TOTDDA** (mmol)	**TREN** (mmol)	**TA** (mmol)
**noXDA**	0.00	3.70	0.20	4.00
**P-XDA 5**	0.20	3.50	0.20	4.00
**P-XDA 7.5**	0.30	3.40	0.20	4.00
**P-XDA 10**	0.40	3.30	0.20	4.00
**P-XDA 12.5**	0.50	3.20	0.20	4.00
**P-XDA 15**	0.60	3.10	0.20	4.00
**P-XDA 17.5**	0.70	3.00	0.20	4.00
**P-XDA 20**	0.80	2.90	0.20	4.00
**P-XDA 30**	1.20	2.50	0.20	4.00
**P-XDA 40**	1.60	2.10	0.20	4.00
**P-XDA 50**	2.00	1.70	0.20	4.00
**XDA+TA**	2.00	0.00	0.00	2.00

2. Experimental setup

2.1 Raman Spectroscopy

Raman spectra were recorded using a WITec Alpha 300R Raman microscope. A 784.8 nm near-infrared laser with Topica XTRA diode was used on conjuncture with the Raman microscope (*Note*: for the linear **TDA+TA** samples the 785 nm laser was used on low intensity, as the samples quickly burned when too high intensity was used). The spectrometer was a UHT300S spectrometer by WITec with a grating of 600 g/mm. The microscope was controlled using a WITec Control FIVE suite, which was also used for initial data processing of the Brightfield and Raman data. Further data processing was performed using the joining WITec Project FIVE software. For Brightfield imaging, Zeiss EC Epiplan-Neofluar objectives were used with a magnification of either 20×, 50× or 100×, and a numerical aperture of 0.5, 0.8 and 0.9, respectively. Raman images were constructed over an area of either 50×50, 100×100, or 150×150 μm, with resolutions of 100x100 pixels. Scale bars are always presented in the bottom left corner of the shown image.

Calibrations and signal intensity checks were performed using a crystalline silicon substrate, for which the Raman signal was set at 520 cm^{-1} as reference value.[S2]

All polymer samples were analyzed by placing the film directly under the microscope. It was then closed from the surrounding environment by placing of a costume-made black screen around it. The screen served two purposes: to block incoming light that could potentially interfere at the detector, and to protect the user from potential reflecting laser light.

2.2 Rheology

Rheology measurements were performed on an Anton Paar MCR501 rheometer, equipped with a temperature-controlled plate-plate geometry and a temperature control hood for additional thermal homogeneity. All rheological measurements were performed on polymer discs with a diameter of 10 mm and a thickness (gap size) of 0.5 mm. Due to thermal expansion the gap size may slightly drift over higher temperature domains. If needed, a normal force control of 1 N could be applied when thermal expansion or shrinking occurred that would deteriorate contact between the sample and geometry.

Temperature sweep experiments were performed with increments of 1 °C every 20 seconds, while the samples oscillated with a frequency of 1 Hz and a strain of 0.1%. Storage- (*G*'') and loss (*G*') were then plotted as a function of the temperature. The tan(δ) was calculated as *G*''/*G*', and was also plotted as a function of the conjunct temperature.

Creep tests were performed by applying a constant stress of 10 kPa to the samples, following the deformation (%) over time up to a total of 1000 s. All creep tests were performed at 20 °C and ambient atmosphere.

2.3 Atomic force microscopy

Atomic force microscopy (AFM) was performed using an Asylum MFP-3D Origin AFM (Oxford Instruments, United Kingdom). The instrument was operated in tapping mode and equipped with a silicon cantilever (AC240TS-R3, $k = 1.3\ \mathrm{N\ m^{-1}}$) with a nominal tip radius of ≈ 7 nm. The acquired images were processed using Gwyddion 2.53 software.

2.4 3D multiphoton fluorescence lifetime microscopy

Multiphoton laser scanning microscopy images were recorded on a Leica SP8 DIVE multiphoton microscope equipped with a Coherent (inc) Chameleon Vision II Ti:Sapphire laser. A 40×/1.1 HC PL IRAPO objective was used for both images. Fluorescence was induced by simultaneous excitation of the sample by two photons (excitation wavelength 720 nm). Emission was recorded between 530–570 nm. The size of each image was 145×145 µm, with a pixel size of 133×133 nm. For a 3D reconstruction, a total of 128 images were recorded in a depth of 80 mm. For optimal results, oil was added between sample and cover glass to alleviate the refraction index mismatch slightly. An overview of all settings is presented in Figure S1 on the next page.

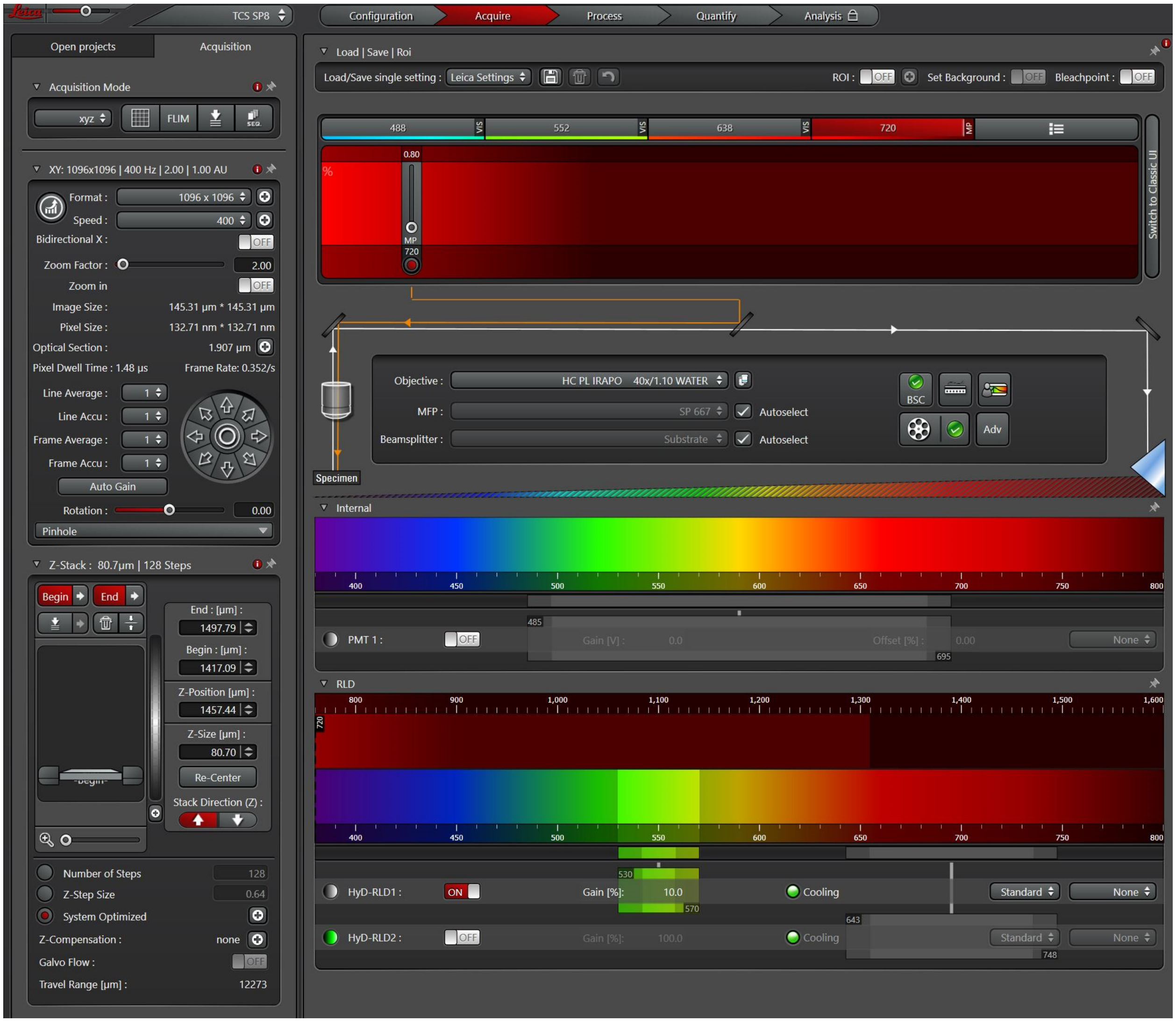

Figure S1. Overview of applied settings for the 3D multiphoton fluorescence lifetime microscopy.

3. Assignment of Raman signals

3.1 Raman spectra of 20% P-XDA samples

The initial full Raman spectra of all five **P-XDA** (20%) materials are shown in Figure S2. Three main regions in the spectra are noted: at 0–200 cm^{-1}, 1100–1250 cm^{-1} and 1500–1700 cm^{-1}. The signals in the region of 0–200 cm^{-1} were assigned to the aliphatic parts of the material caused by the presence of **TOTDDA** and **TREN**, and were found to be of minor interest compared to the other two regions that included information on the aromatic parts and imine groups. Therefore, the following Raman spectra will be shown only from 1000–1750 cm^{-1}.

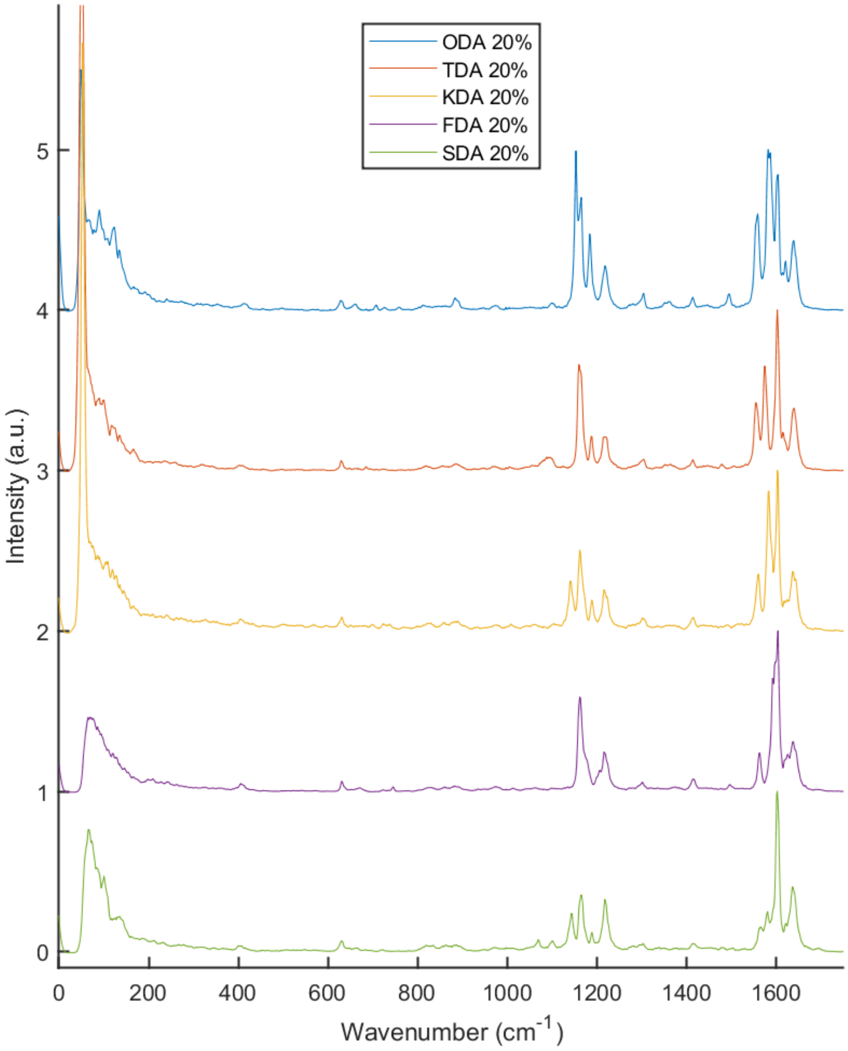

Figure S2. Full Raman spectra of 20% **P-XDA** materials. From top to bottom: **P-ODA** (blue), **P-TDA** (red), **P-KDA** (yellow), **P-FDA** (purple), **P-SDA** (green). All spectra showed main signal regions at 0–200 cm^{-1}, 1100–1250 cm^{-1} and 1500–1700 cm^{-1}.

3.2 Raman spectra of **NoXDA** and **XDA+TA** analogue samples

To accurately assign all Raman signals, analogue materials were synthesized in addition to the **P-XDA** (20%) samples. These included a **NoXDA** sample, which contained all monomers except the **XDA**, and linear **XDA+TA** samples, which contained only the **XDA** and **TA** monomers. Overlaying the Raman spectra of **NoXDA** and **XDA+TA** resulted in a good resemblance of the full **P-XDA** spectra (Figure S3), and facilitated spectrum assignment. Together with literature data on other analogue materials (see Table 1 of the main text), each signal was allocated to a part in the material. The full assignment of signals is discussed in more detail in the following paragraphs.

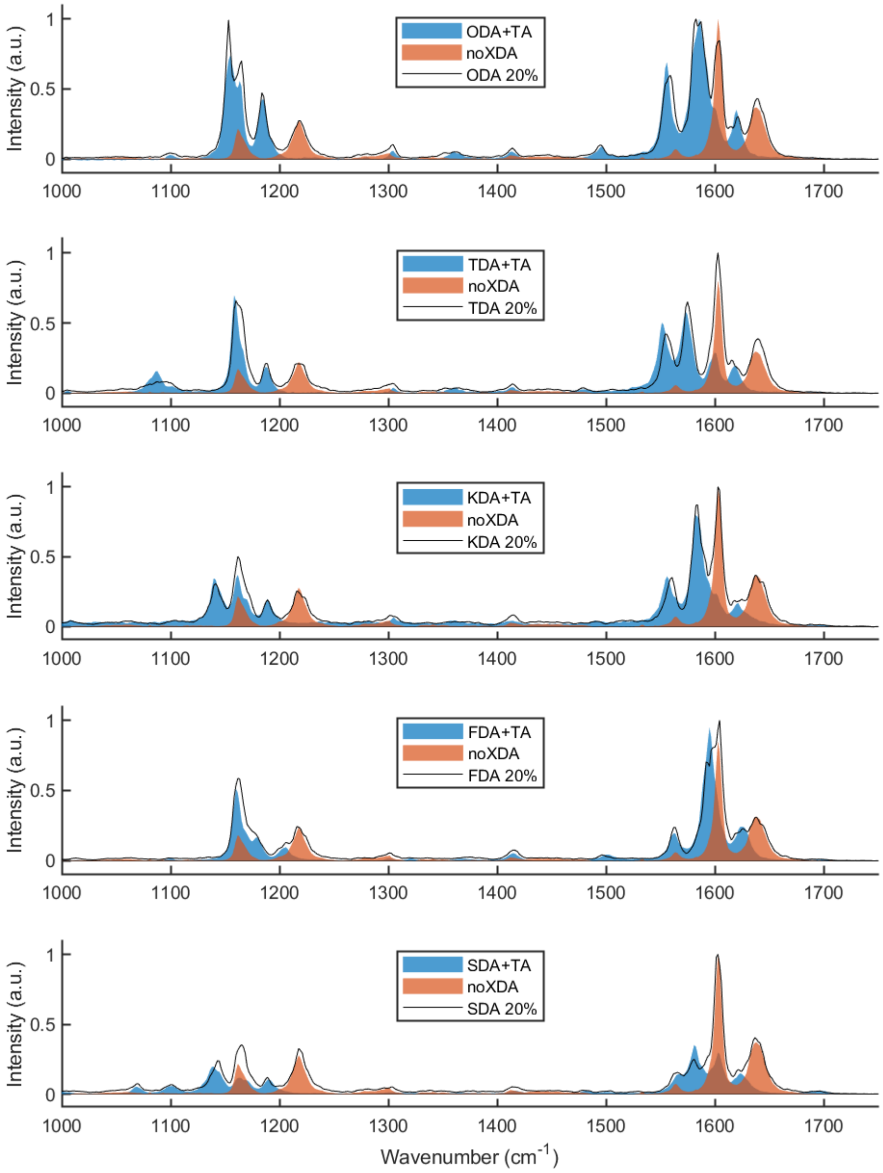

Figure S3. Overlayed Raman spectra of **XDA+TA** (blue) and **NoXDA** (orange) analogues to show the resemblance to the full **P-XDA** spectrum (grey line).

3.3 Common signals from 1100–1250 cm^{-1}

Two common peaks in this region were found for all samples at approximately 1165–1170 cm^{-1} and 1215–1225 cm^{-1}. Since these signals were common to all samples, they were expected to be caused by the parts of **TOTDDA**, **TREN**, and **TA**. This was confirmed from the from the Raman spectrum of **NoXDA** (Figure S4), containing each of the three mentioned monomers. From documented literature on similar geometries the signal at 1163 cm^{-1} could be assigned to phenyl C–H bend vibrations of the **TA** parts, and the signal at 1218 cm^{-1} could be assigned to imine C–N stretch vibrations from the **TOTDDA** and **TREN** parts.[S3-4]

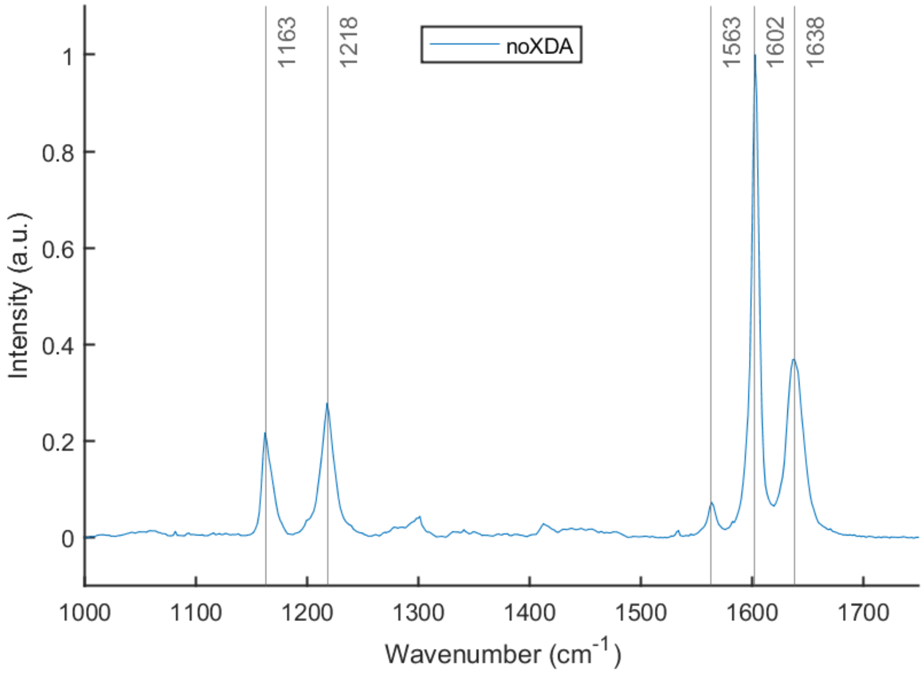

Figure S4. Raman spectrum of **NoXDA** from 1000-1750 cm^{-1}.

3.4 Common signals from 1500–1700 cm^{-1}

In this region, signals for aromatic rings, imines and aldehydes are commonly found.[S5] In the polyimine samples, there are two clear peaks common to all samples; a strong signal at 1602 cm^{-1} and a medium signal at 1638 cm^{-1}. Additionally, a small signal at 1563 cm^{-1} could be observed for **NoXDA**, which generally overlapped with a much stronger signal from the **XDA** containing samples. From reported literature, the signals at 1563 and 1602 cm^{-1} could be assigned to aromatic C=C stretch vibration, and the signal at 1638 cm^{-1} to imine C=N stretch vibrations.[S3-6]

3.5 Sample specific signals from 1100–1250 cm^{-1}

For each sample, at around 1190 cm^{-1}, there is a signal which corresponds to the C–N signal close to the dianiline monomer. This conclusion is based on research by Nandi *et al.* (2017),[S3] where a similar C–N bond between an imine group and an aromatic ring caused a signal with a wavenumber of 1190 cm^{-1}.

Next, a sample-specific signal in the region 1140–1160 cm^{-1} is observed. For **P-ODA**, **P-TDA** and **P-FDA** this signal appears at 1153 cm^{-1}, 1158 cm^{-1} and 1160 cm^{-1}, respectively, while for **P-KDA** and **P-SDA** the signal appears at approximately 1140 cm^{-1}. In this region, the C–H bending vibration for aromatic moieties are generally observed.[S3-4, 7-8] As such, these signals were assigned to the phenyl C–H bend vibrations of the tunable **XDA** monomer parts.

3.6 Sample specific signals from 1500–1700 cm^{-1}

In this region, the expected signals consist of (I) the C=C stretch vibrations for the dianiline phenyls, (II) the **TA** signal noted before and (III) the imine signal for the imine between dianiline and **TA**.

The **TA** signal was once again found around 1602 cm^{-1}, and assigned accordingly. Furthermore, a signal at around 1620 cm^{-1} is observed. This signal is assigned to the imine between the **XDA** and **TA** monomer, specifically to the C=N stretching vibration. For compounds with similar geometries (two phenyl rings with an imine in between),[S3-4] the imine signal was also observed around 1620 cm^{-1}. Moreover, it is relatively close to the previously established imine signal for the imines between an aliphatic chain and **TA**, but shifted to slightly lower wavenumbers. Due to conjugation to the aromatic system, and thus being slightly more stable, this is an expected position for the imine signal. Additionally, for each sample, there are two signals in the region from 1550 cm^{-1} to 1600 cm^{-1}. These signals are likely caused by the **XDA** parts, at least one being caused by the C=C stretch vibrations of the phenyls in the **XDA**. Research by Ullah *et al.* (2019)[S8] reviewed the Raman spectra of unreacted **SDA**, both using Density Functional Theory (DFT) calculations and by experimental results. They found two signals, at 1584 cm^{-1} and 1602 cm^{-1} which both corresponded to the C=C stretch in the phenyls. It is thus not unlikely that a similar split into two signals is possible for the rest of the dianiline monomers.

4. Raman image analysis for fitting with a binary scale

As mentioned in the main text of this study, phase separation for **P-TDA** materials was observed when the dianiline content was at least 15% of the total amount of amines. This was confirmed by Raman imaging of several samples with varying dianiline content. The samples with a dianiline content of 12.5% and below did not show a clear phase separation, but their binary Raman images still showed an apparent division in blue and yellow domains. This was the result of the (built-in) fitting procedure within the WITec software that for each pixel enforced an assignment to either blue or yellow, in accordance with which reference spectrum the measured spectrum fitted best (*i.e.*, yellow for a best fit to **TDA+TA**, and a blue color to a best fit to **NoXDA**). However, by comparing the spectra of a yellow and blue domain, it is possible to check if the two regions indeed show different Raman spectra. As an example, Raman images of **P-TDA 10** are presented in Figure S5. First, a Raman image was constructed from a best fit between reference spectra of **TDA+TA** and **NoXDA** (Figure S5A). Then, for both Raman images the spectra of randomly chosen blue and yellow pixels were called up and shown (Figure S5B). The Raman spectra of the selected yellow and blue pixels appeared highly similar, suggesting that no phase separation was present, but that the experimental spectrum is such a composite of the two reference spectra that in one case it just so slightly more represents the **TDA+TA** ('yellow') spectrum, and in the other case the **NoXDA** ('blue') spectrum, leading to the artificial contrast. This double check was performed for all other Raman images as well. As such, we could always determine retroactively if an observed phase separation profile was justified. For comparison, the Raman image and spectra of blue and yellow color attribution of phase-separated **P-TDA 20** are shown in Figure S6. Here, it can be seen that the Raman spectra for yellow and blue pixels indeed resemble those of the reference **TDA+TA** and **NoXDA** spectra. As such, we can claim that the observed phase-separated profile is real.

An additional check to confirm whether phase separation could be observed or not, is by using a continuous scale for fitting. This will be discussed in the following paragraph.

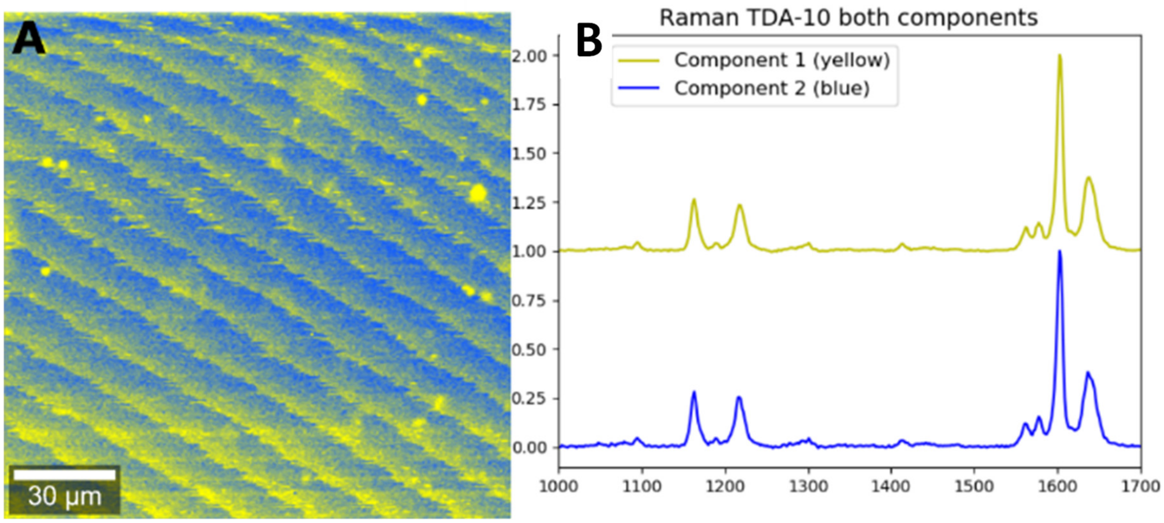

Figure S5. A) Raman image of **P-TDA 10**, and B) Raman spectra of a random yellow and blue pixel. The Raman spectra indicate that there is no clear difference between yellow and blue pixels in the Raman image, meaning that no phase separation was present.

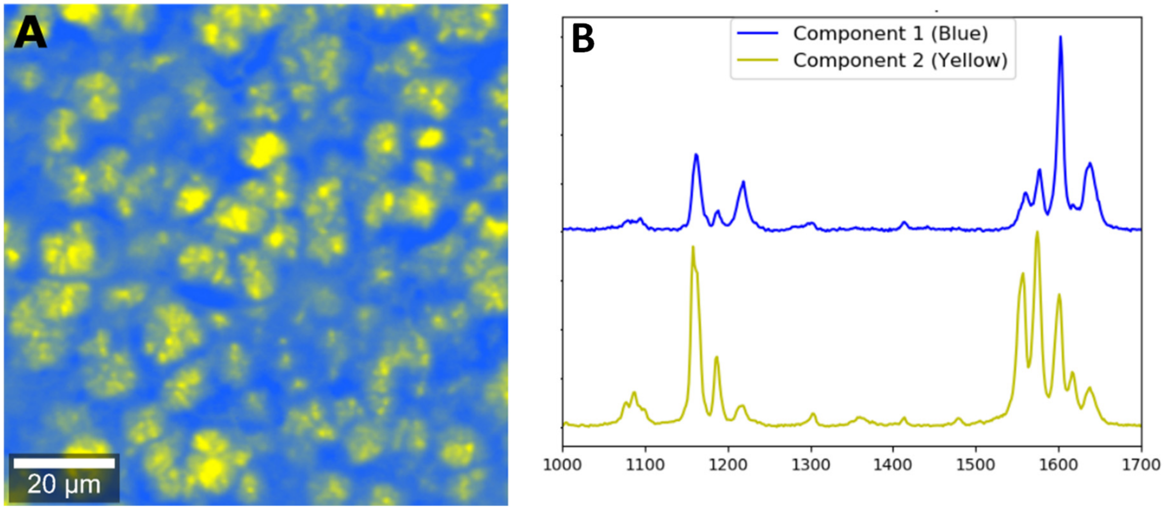

Figure S6. A) Raman image of **P-TDA 20**, and B) Raman spectra for a random blue and yellow pixel. The Raman spectra show proper identification of different spectra that resemble either that of reference **TDA+TA** (yellow) or **NoXDA** (blue). As such, the observed phase-separated profile is real.

5. Continuous coloring scale of Raman images

To overcome the problems regarding the ‘binary’ pixel assignment (in either ‘yellow’ or ‘blue’), we chose to manually fit and analyze selected spectra on a continuous scale. More specifically, we used it to reduce the artificial contrast observed in some of the Raman images (see Section 4). A 256-point scale (yellow-red-black) was applied for each pixel in the Raman image based on the ratios of peak areas between the different signals. Relying on peak area ratio rendered our analyses insensitive to variations in laser intensity. Images are now constructed that reveal more about the actual amounts of either the aromatic (**TDA+TA**) and aliphatic (**NoXDA**) composition, rather than an ‘all-or-nothing’ binary fit. This method also proved efficient to remove artifacts in the Raman images. As an example, in Figure S7 three binary-scale Raman images are presented of A) a phase-separated material, B) a non-phase-separated material giving rise to a noisy Raman image, C) a non-phase-separated material with artifacts (diagonal lines appear over the image). In Figure S8, the same images are presented, but now on the continuous scale. Here, we can clearly see that the observed phase separation in panel A can be confirmed, and that the phases consist of high amounts of the individual components. We also see that the noisy non-phase-separated material (in panel B) indeed shows no phase separation, as the continuous scale shows a mostly well-blended image. Last, we see that the artifacts in panel C are now fully removed to show a non-phase-separated image.

The continuous-scale fitting can thus serve as a more reliable check to confirm whether phase separation was indeed real and not the result of artifacts. For routine checks, we do however still use the binary fitting as a quick method to see if phase separation is present, as it generally shows a better contrast between the borders of the (two) domains and saves time due to its simple setup (and availability in the software). The continuous-scale fit can later serve as either a check to confirm what was observed before, or to clear up artifacts or otherwise unclear images. As such, in the main manuscript we provided both binary and continuous-scale images, whereas the majority of additional images in the Supporting Information will only show the binary fitting.

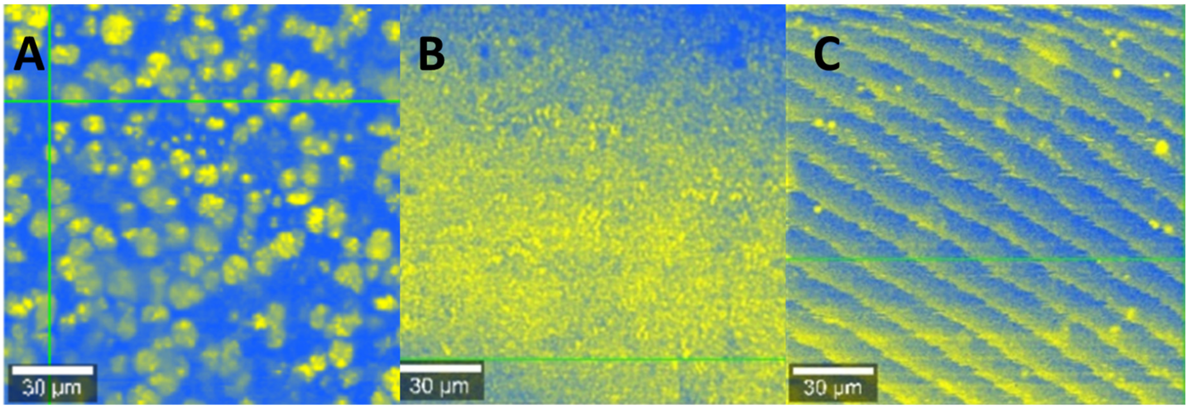

Figure S7. Binary scale Raman images of A) a phase-separated material, B) a non-phase-separated material giving rise to a noisy image, C) a non-phase-separated material with artifacts as diagonal lines over the image.

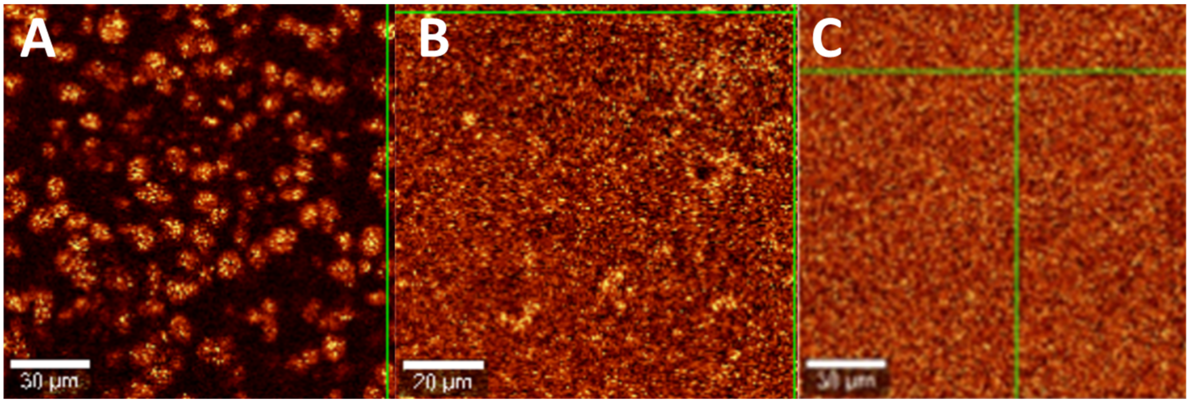

Figure S8. Continues scale Raman images of the same materials presented in Figure S7. Here we see that the phase-separation can be confirmed for A, that B did indeed not phase-separate, and that the artifacts in C can be removed to show that indeed no phase separation occurred.

5.1 Procedure for creation of continuous-scale Raman images

To construct the continuous-scale images the following protocol was executed in the Project FIVE software:

1. Load the data files.
2. Hold right click on the data file (most likely Scan Piezo), and select the lightning icon (data analysis).
3. Use the filter option.

4. Create filters using the screen below:

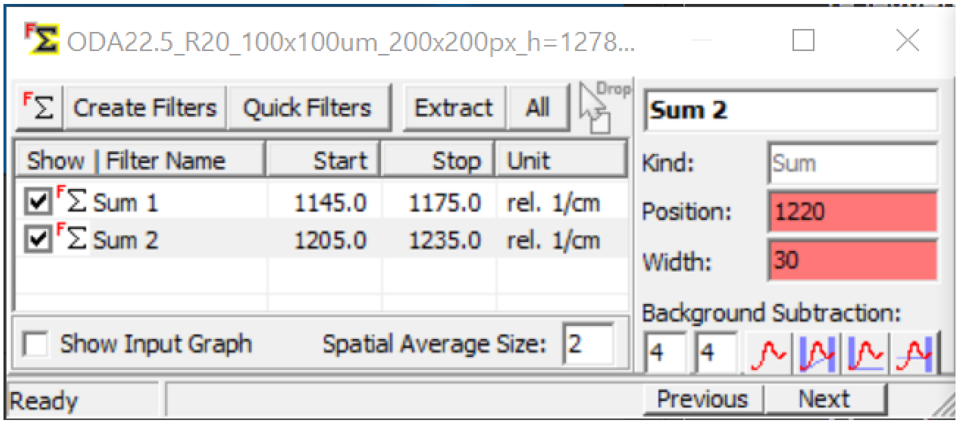

5. In the right box, put the position in the middle of the desired peak(s), and adjust the width until the entire peak range is covered. The spectrum will look something like the example below:

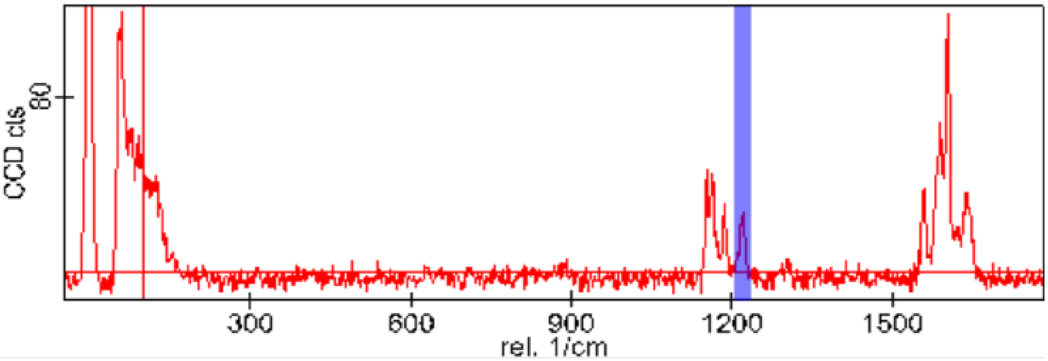

6. In the window shown in step 4, tick all boxes of the spectra and then click on the "All" button in the right corner. This will create a new dataset from the specific peaks.
7. In "Project Manager" select both spectra and hold right click on the files, and again select data analysis.
8. This time, click on "Calc", and a new window will open, looking similar to the one below:

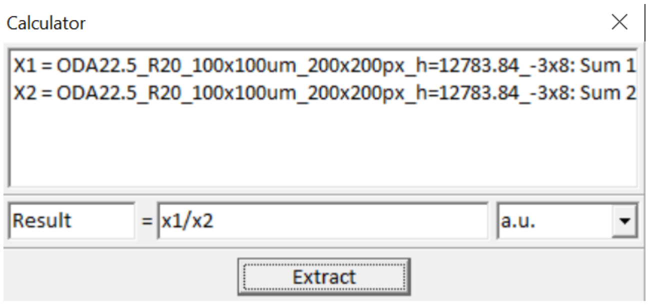

9. Use the X1 and X2 data files to calculate a new data file using the extract button below. Loading this datafile will prompt a new Raman image showing the continuous-scale fit.

6. Fitting without reference spectra and ruling out a third phase

In the main manuscript we mentioned that the separation of phases was always carried out into two phases (the **XDA+TA** phase and **NoXDA** phase). We were able to do this because we could obtain reference spectra of the corresponding polymer parts. In case no reference spectra are available, or if it is not known what the chemical composition of the separated phases is, the WITec Project FIVE software enables an automatic component analysis method, which separates the image in different components. This way, two, or more, phases can be automatically generated. And by retroactively checking the Raman spectra of the ascribed pixels (as mentioned in Chapter 4 of the ESI) it is possible to see if the ascribed colors indeed represent chemically different domains. We applied this automatic fitting to check if these images would show the same outcome as when fitted using reference spectra. Second, we used the method to check if potentially a third (or more) phase(s) were present. As an example, Raman images of **P-TDA 17.5** are shown in Figure S9 to better explain these features, and substantiate our chosen method for visualization of the phase separation by Raman imaging.

Figure S9A first shows a Raman image of **P-TDA 17.5** that was constructed by fitting with the reference spectra of **TDA+TA** (yellow) and **NoXDA** (blue). A clear phase separation was observed, and also by inspecting full spectra for individual pixels, we could see that the yellow and blue pixels are correctly ascribed to the corresponding phases.

Next, Figure S9B shows the same Raman image, however, it is now fitted into two phases using the automated fitting function of the WITec Project FIVE software. The same phase-separated profile between yellow and blue domains is made visible, however, also darker areas are shown. We then called the Raman spectra for yellow and blue pixels again, but this time we also checked what represented these darker domains. The Raman spectra of yellow and blue pixels gave good agreement to the reference spectra that we used before. The Raman spectra of the darker areas gave similar Raman spectra as the reference spectra, however, with a much higher signal-to-noise ratio.

Last, Figure S9C shows the same Raman image, but fitted into *three* phases using the automated fitted function. We then obtained an image with red, green and blue colors. By checking the Raman spectra of the ascribed colors, we found that the blue color was in good coherence to the **NoXDA** reference, and the red color showed good coherence to the **TDA+TA** reference (before always shown as yellow). The separation between blue and red appeared similar to the images obtained

before, but now green shades appeared, which could potentially indicate a third phase. However, we found that the green color resembled a mixed phase between **TDA+TA** and **NoXDA**, ruling out the presence of a third phase.

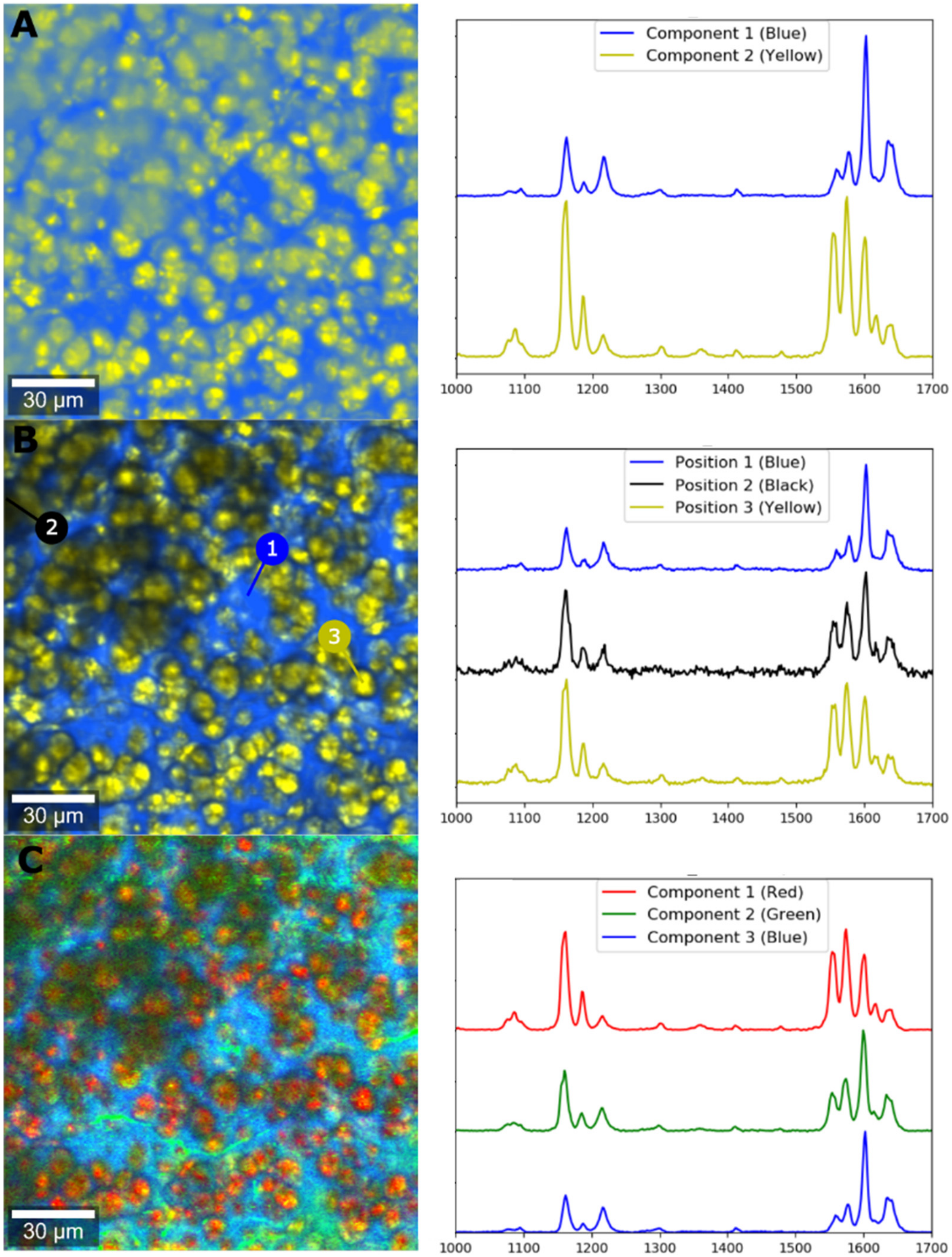

Figure S9. Raman images of **P-TDA 17.5**, which were constructed by A) fitting with reference spectra of **TDA+TA** and **NoXDA**, B) using the automatic fitting in *two* components, C) using the automatic fitting in *three* components. On the right of each Raman image spectra are shown of corresponding pixels in that color.

7. Depth scans of 15 and 20% P-TDA samples

Depth scans of **P-TDA** samples with 15% and 20% dianiline contents are shown in Figure S10 and Figure S11, respectively.

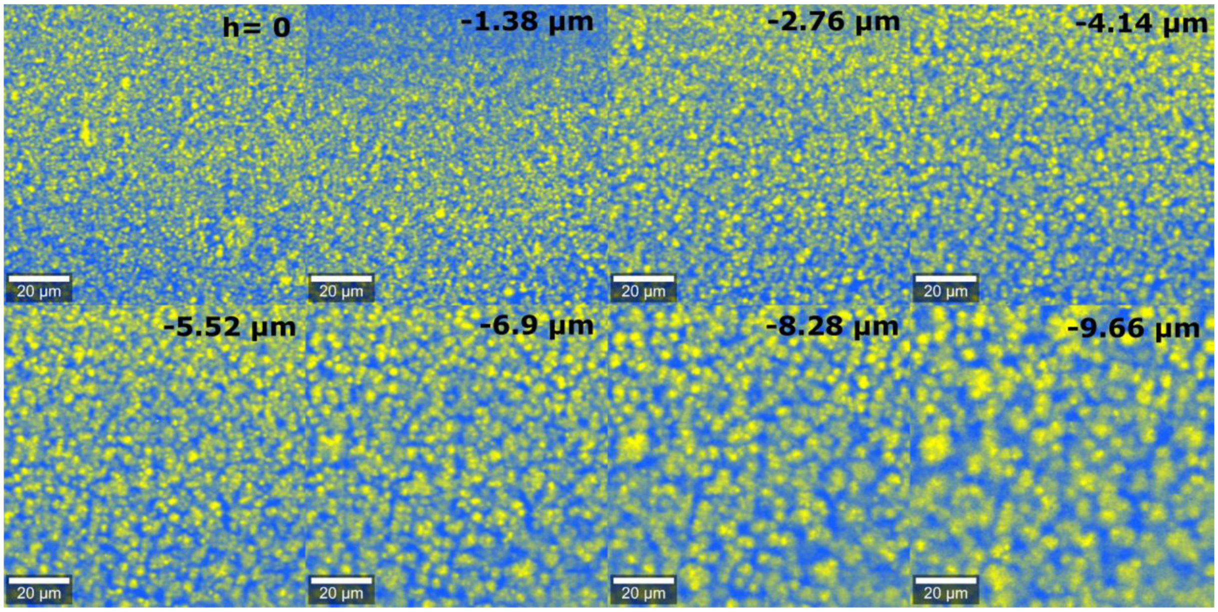

Figure S10. Raman images of **P-TDA 15** at various depths from the surface. Domains of **TA+TDA** (in yellow) appear to increase in size at lower depths (deeper into the sample when moving from the surface).

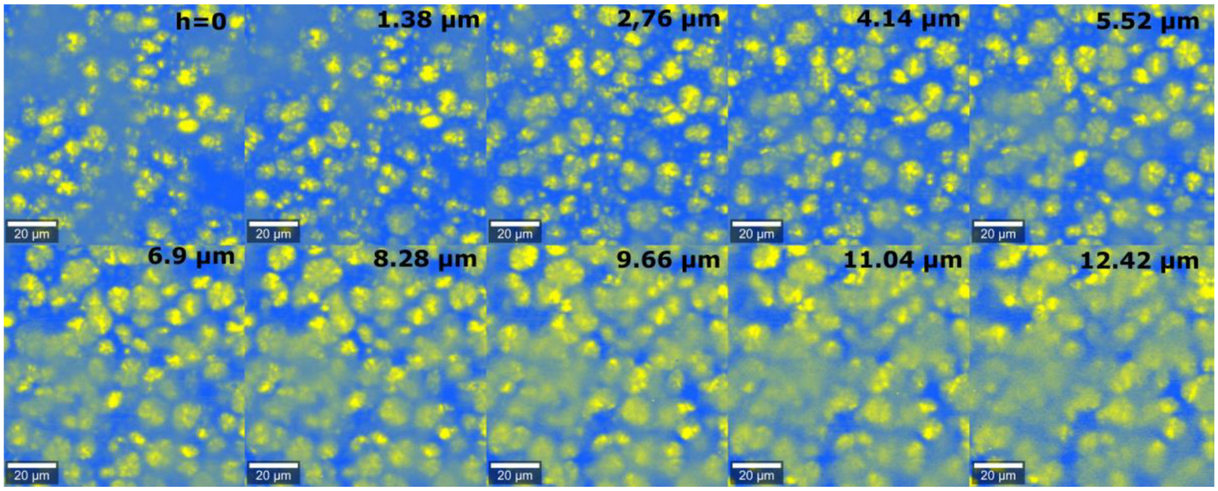

Figure S11. Raman images of **P-TDA 20** at various depths from the surface. Domains of **TA+TDA** (in yellow) appear to increase in size at lower depths (deeper into the sample when moving from the surface).

8. Brightfield and Raman images of P-TDA20 films with different thickness

To investigate if the sizes of the phase-separated domains were affected by the thickness of the material, **P-TDA 20** films with different thicknesses were prepared. The default synthetic preparation[S1] of ~1 gram of polymer results in films with a thickness of 0.5 mm when a petri dish with a radius of 4.5 cm is used. Therefore, thinner samples were now prepared of 0.1 mm and 0.2 mm thickness by using the same size petri dish, but only pouring 20% and 40% (respectively) of the prepolymer material into the petri dish. Also, a sample was prepared from only one drop of the prepolymer solution. Brightfield and Raman images of these samples were obtained (Figure S12). The images show that the size and distribution of the microdomains in all thin films were similar to that of the default 0.5 mm thick sample. This thus suggest that the observed phase separation at the surface of the polymer film is not affected by the scale of the preparation.

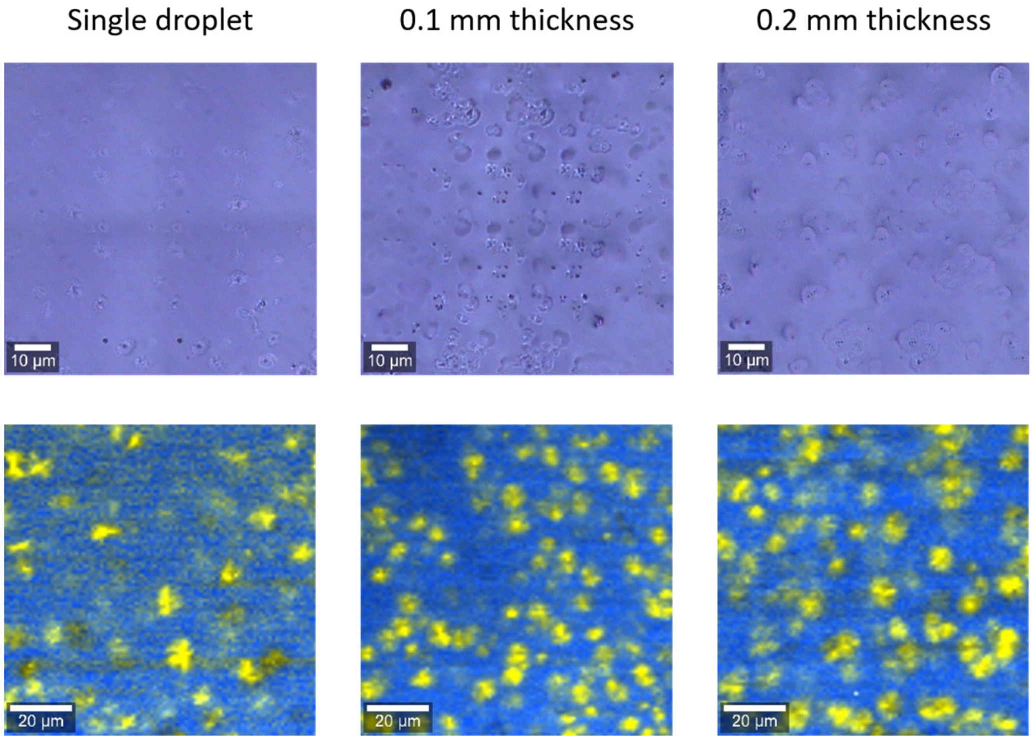

Figure S12. Brightfield (top) and Raman (bottom) images of **P-TDA 20** with different film thicknesses. The images in the first column (left) are from a single drop, the second column (middle) are of a film with a thickness of 0.1 mm, and the third column (right) are from a film with a thickness of 0.2 mm.

9. Raman images of P-TDA20 samples, synthesized from different solvents

The Brightfield and Raman images of **P-TDA20**, synthesized from various solvents, are presented in Figure S13 below. From left to right: MeOH, 2-MeTHF, EtOAc and toluene. The phase separation can be observed for all samples, although it remains difficult to make a clear distinction between the different patterns regarding size or distribution. Note the different scale bars for comparison.

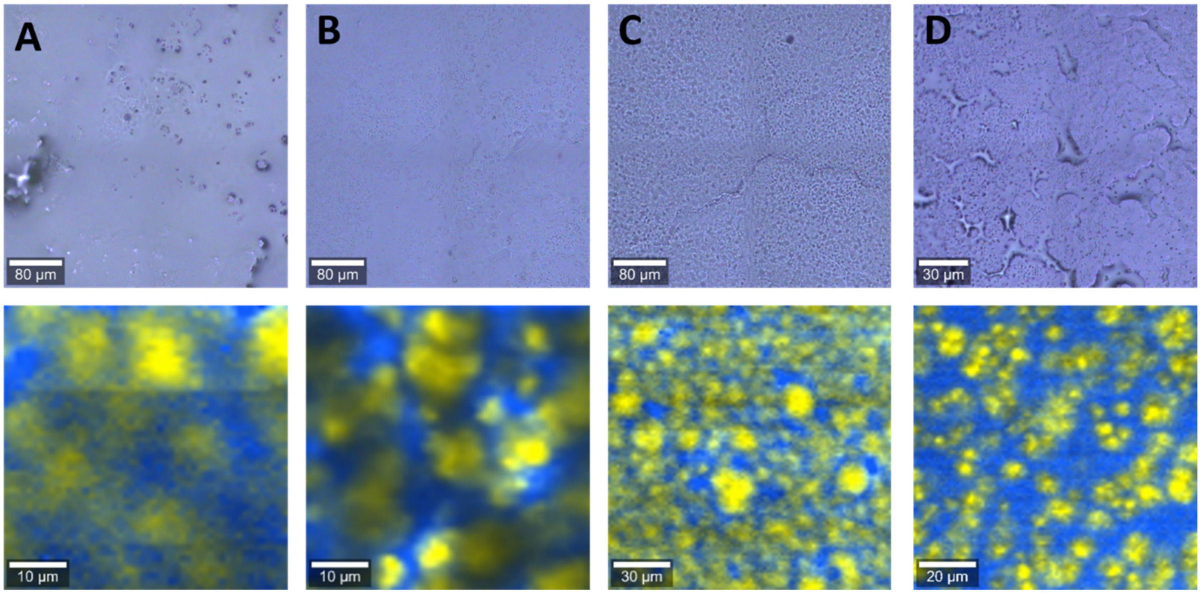

Figure S13. Brightfield (top) and Raman (bottom) images of **P-TDA 20**, synthesized using different solvents. A) MeOH, B) 2-MeTHF, C) EtOAc, D) toluene. Images were recorded of dried materials after the synthesis, without any additional hot-pressing or other (re)reprocessing steps.

10. Dynamic Light Scattering (DLS)

To investigate if microdomains would already be formed in solution during the polymerization reaction, we used dynamic light scattering (DLS). We analyzed two phase-separating materials (**P-TDA 20** and **P-MDA 20**) and two non-phase-separating materials (**P-SDA 20** and **P-MeMDA 20**). For each material two solutions were prepared: an aldehyde solution of **TA** (13.4 mg, 0.100 mmol) in THF (1 mL), and an amine solution of the corresponding **XDA** (0.020 mmol), **TOTDDA** (15.9 μL, 0.0725 mmol) and **TREN** (0.75 μL, 0.005 mmol) in THF (1 mL). The two individual solutions were first filtered, and then examined by DLS to check that no impurities of larger size were present. Then, the two solutions were mixed together and DLS was used to follow over time if clustered particles would form. A scan was performed every 30 s for a total duration of at least 18 hours, and the hydrodynamic radius of detected particles was plotted as a function of the time. Additionally, the median radius for every 30 min was plotted.

For the phase-separating materials, a remarkable difference between **P-TDA 20** and **P-MDA 20** was observed. For **P-TDA 20 (**Figure S14), particles sizes were mainly clustered around a size of 1 μm, which started to grow after roughly 5 hours. For **P-MDA 20** (Figure S15) a much different trend was observed in which at the start larger particles were mainly observed around 100 μm, which over time started to decrease to the range of several microns. For the non-phase separating materials the mean particle size did not seem to grow or shrink over time. However, with time the size distribution became larger. For both **P-SDA 20** (Figure S16) and **P-MeMDA 20** (Figure S17), particles sizes were typically observed in the range from 10–100 μm for roughly the first 10 hours, but after that more particles with sizes below 10 μm were observed. It remained unclear what the cause for the observed trends were, and it was difficult to translate the solution dynamics over these elongated time intervals to the synthetic process of the polymer films. This is mainly due to the fact that during the normal preparation of polymer films most solvent is evaporated within several hours, leading to different solvation and drying dynamics. More in-depth kinetic analyses of cluster formation in solution and during drying might therefore form the basis of future research.

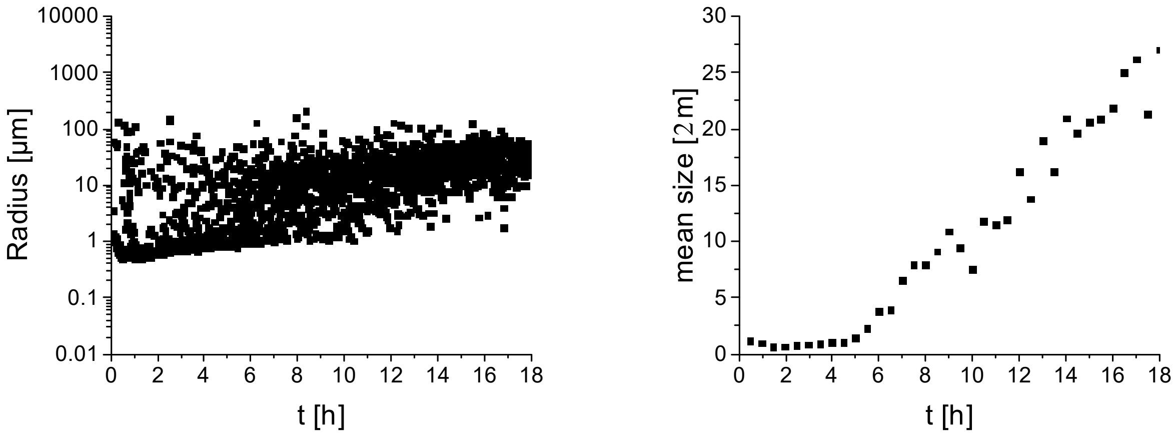

Figure S14. Left: DLS plot of particles sizes that are being formed during the synthesis of **P-TDA 20** in solution. Right: Mean hydrodynamic radius over time, binned per 30 min.

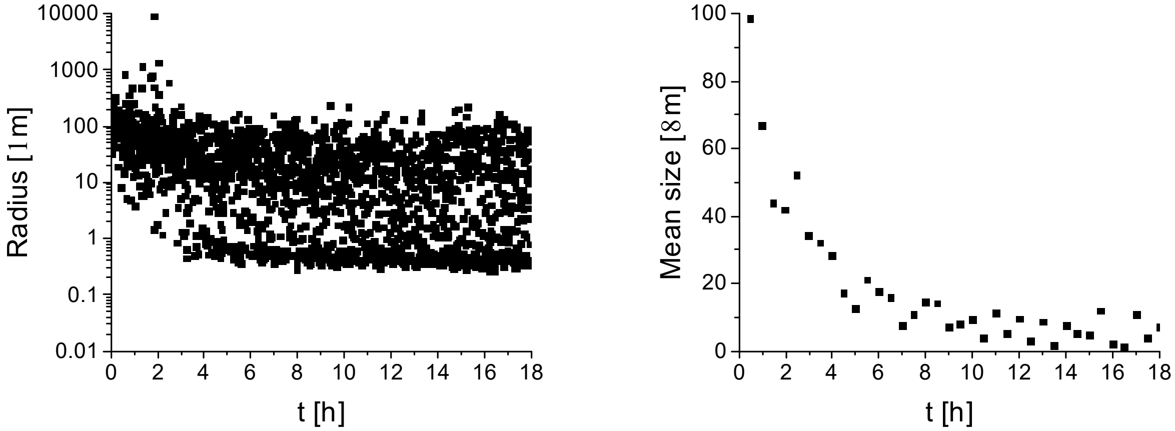

Figure S15. Left: DLS plot of particles sizes that are being formed during the synthesis of **P-MDA 20** in solution. Right: Mean hydrodynamic radius over time, binned per 30 min.

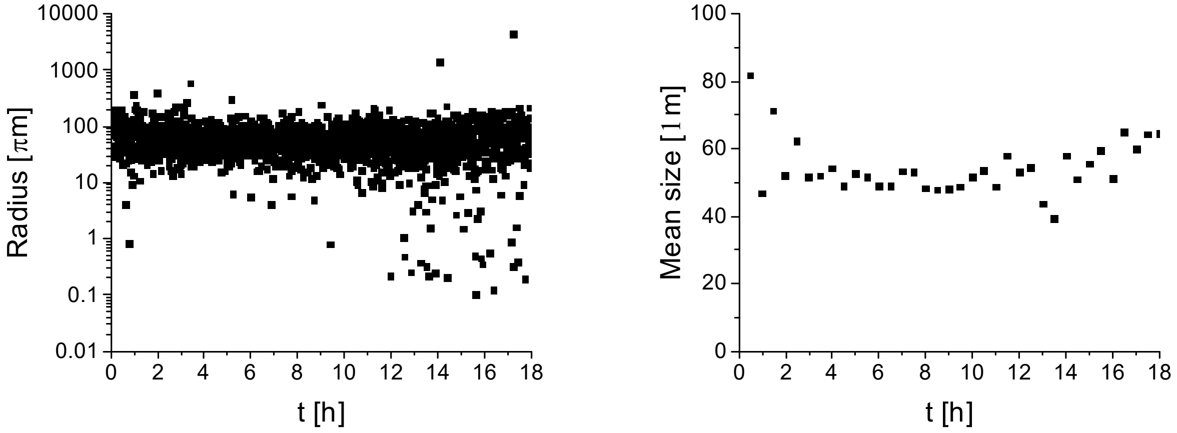

Figure S16. Left: DLS plot of particles sizes that are being formed during the synthesis of **P-SDA 20** in solution. Right: Mean hydrodynamic radius over time, binned per 30 min.

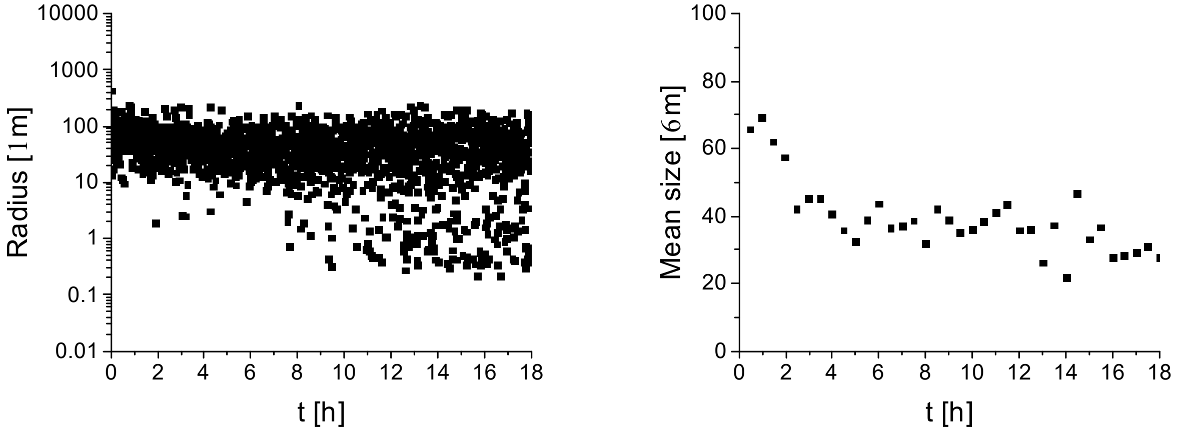

Figure S17. Left: DLS plot of particles sizes that are being formed during the synthesis of **P-MeMDA 20** in solution. Right: Mean hydrodynamic radius over time, binned per 30 min.

11. Material properties of P-TDA samples with dianiline contents from 5 to 20%

For the series of **P-TDA** materials with dianilines contents of 5 to 20% the crossover temperature (T_{cross}), creep after 1000s of 10 kPa stress (γ), and elastic modulus at room temperature (G'_{RT}) are presented below in Table S3 with their corresponding error margins.

Table S3. Material properties of **P-TDA** samples with dianiline contents from 5-20%.

**Material**	**P-TDA 5**	**P-TDA 7.5**	**P-TDA 10**	**P-TDA 12.5**	**P-TDA 15**	**P-TDA 17.5**	**P-TDA 20**
$\boldsymbol{T_{cross}}$ **[°C]**	48 ± 3	53 ± 2	59 ± 2	60 ± 3	59 ± 2	71 ± 1	118 ± 3
$\boldsymbol{\gamma}$ **[%]**	1925 ± 105	751 + 83	362 ± 15	258 ± 90	130 ± 43	20 ± 7	6 ± 1
$\boldsymbol{G'_{RT}}$ **[MPa]**	0.25 ± 0.01	0.47 ± 0.03	0.51 ± 0.07	1.1 ± 0.1	2.6 ± 0.2	4.1 ± 0.1	9.4 ± 0.1

12. Photos of P-XDA sample with regards to transparency

Figure S18 below shows a picture of all **P-TDA** samples with dianiline contents from 5 to 20% directly after the synthesis. It can be seen that all samples below 12.5% **TDA** appear transparent and samples above 15% **TDA** appear turbid. The rough surface of **P-TDA 17.5** was caused by air bubbles that escaped during the drying process. Figure S19 show the difference in transparency for phase-separated **P-MDA** and non-phase-separated **P-MeMDA**.

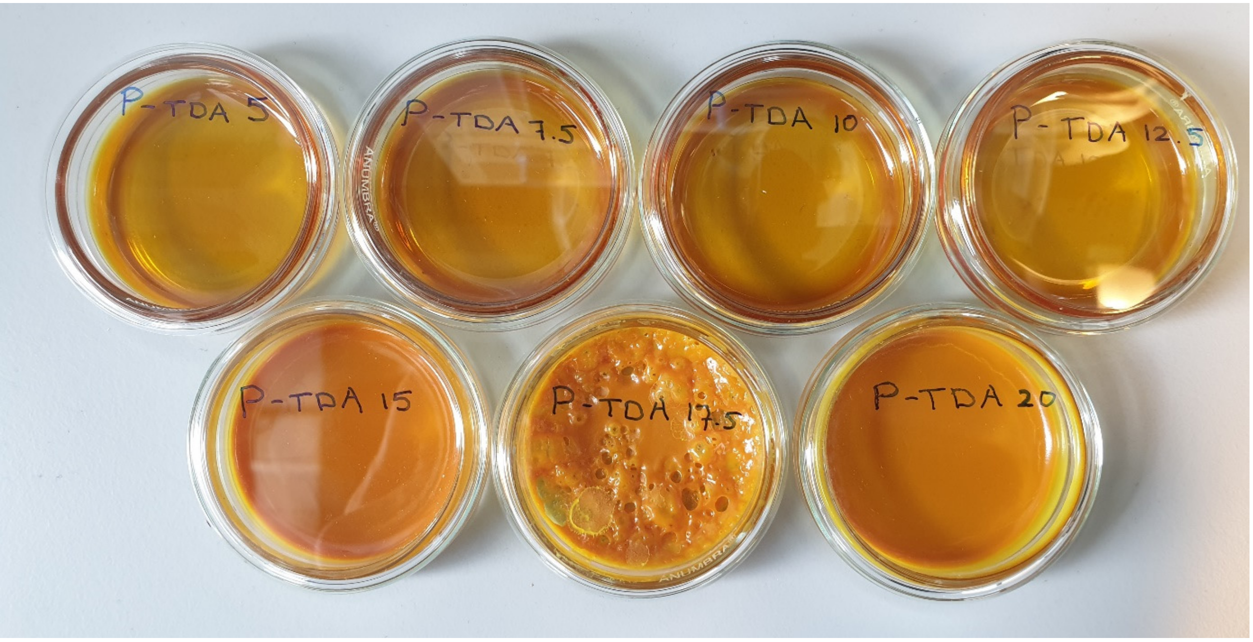

Figure S18. Photo of **P-TDA** samples with 5-20% dianiline contents. Photo was taken directly after the synthesis (including drying, but excluding any additional hot-pressing).

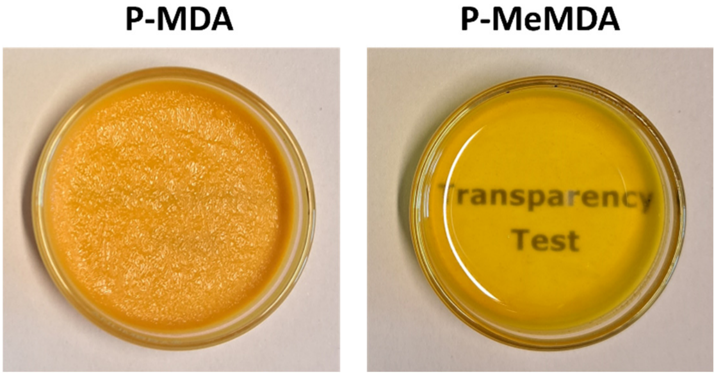

Figure S19. Photo of (left) phase-separated turbid **P-MDA** and (right) non-phase-separated transparent **P-MeMDA**.

13. Temperature sweep curves of P-TDA 5-20

All T sweeps for the **P-TDA** samples were performed *in duplo* and are shown in Figures S20–S26. A linear temperature ramp was set to heat with 1 °C per 20 seconds. A strain of 0.1% with a frequency of 1 Hz was applied. *G*' (blue squares) and *G*'' (red dots) were plotted as a function of the temperature. Tan(δ) was calculated as *G*''/*G*', and was then plotted in the same figure (grey triangles). A horizontal line where tan(δ)=1 was added to determine T_{cross}.

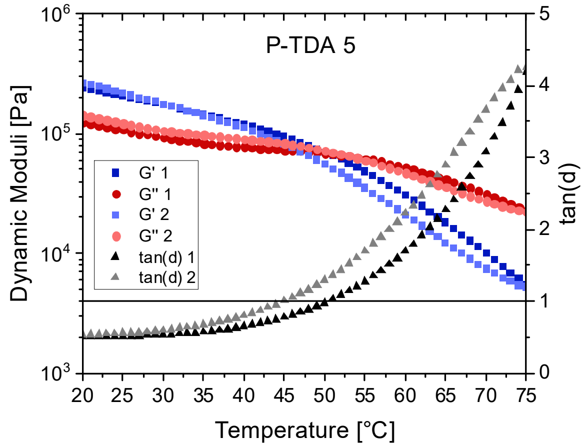

Figure S20. T sweeps of **P-TDA 5**.

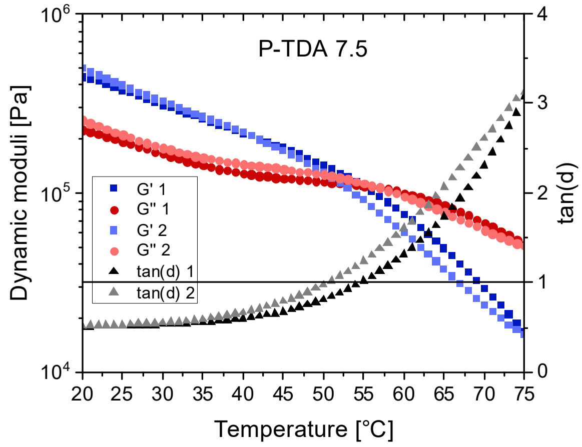

Figure S21. T sweeps of **P-TDA 7.5**.

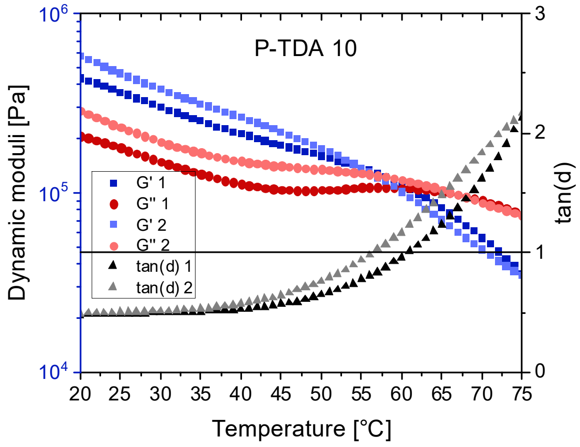

Figure S22. T sweeps of **P-TDA 10**.

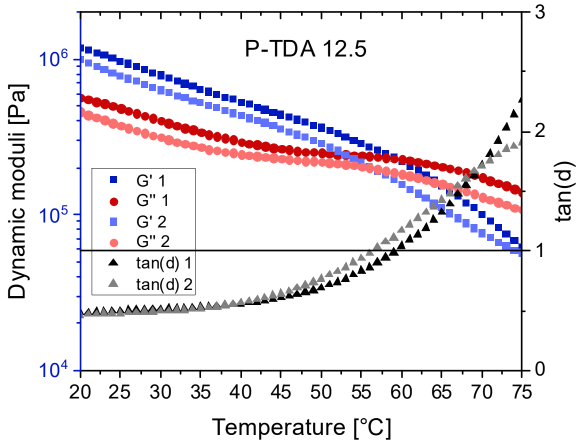

Figure S23. T sweeps of **P-TDA 12.5**.

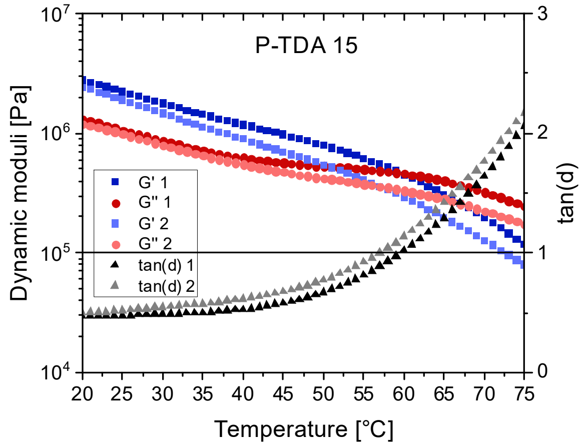

Figure S24. T sweeps of **P-TDA 15**.

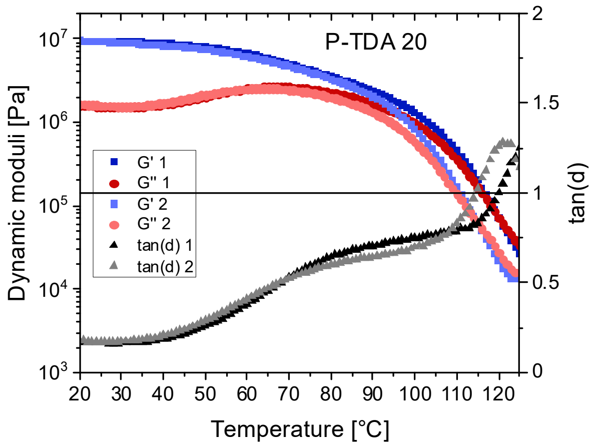

Figure S26. T sweeps of **P-TDA 20**.

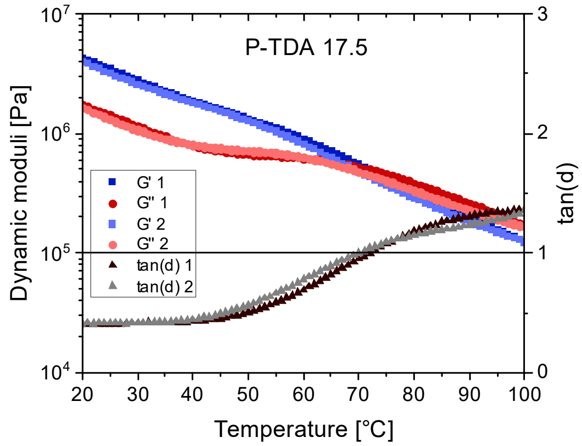

Figure S25. T sweeps of **P-TDA 17.5**.

14. Temperature sweeps of P-MDA, P-MeMDA and P-EtMDA

T sweeps for **P-MDA**, **P-MeMDA** and **P-EtMDA** were performed *in duplo* and are shown in Figure S27, Figure S28, and Figure S29, respectively. A linear temperature ramp was set to heat with 1 °C per 20 seconds. A strain of 0.1% with a frequency of 1 Hz was applied. *G*' (blue squares) and *G*'' (red dots) were plotted as a function of the temperature. Tan(δ) was calculated as *G*''/*G*', and was then plotted in the same figure (grey triangles). A horizontal line where tan(δ)=1 was added to determine T_{cross}.

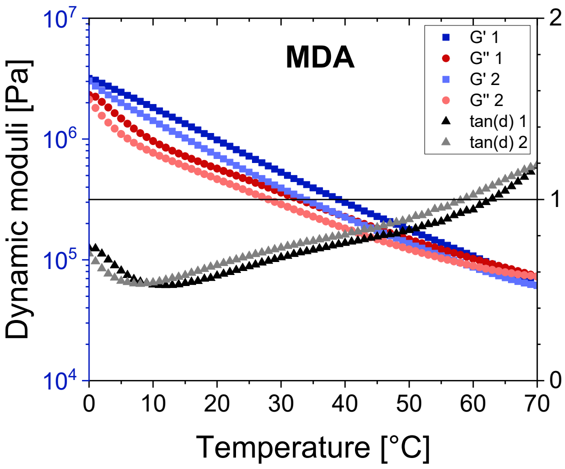

Figure S27. T sweeps of **P-MDA**.

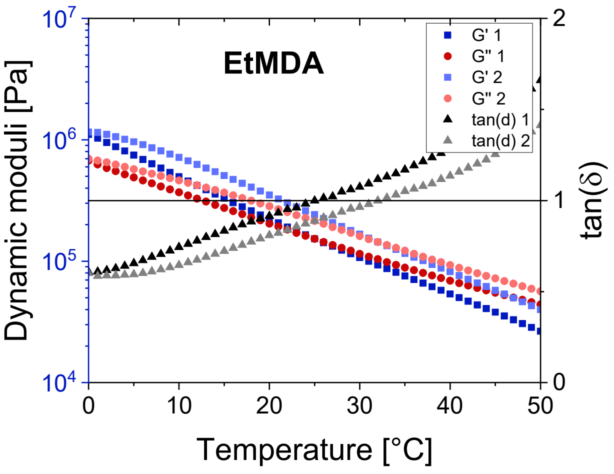

Figure S29. T sweeps for **P-EtMDA.**

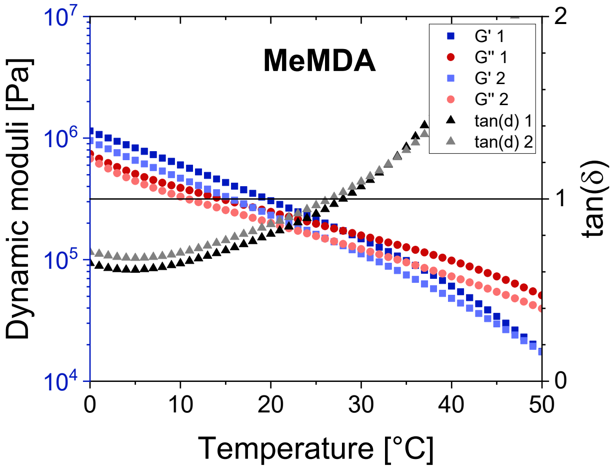

Figure S28. T sweeps of **P-MeMDA.**

15. DSC curves of P-TDA 10 and P-TDA 20

The Differential Scanning Calorimetry (DSC) curves for **P-TDA 10** (red) and **P-TDA 20** (blue) are depicted in Figure S30. Two cycles were measured for each material. Both materials show a similar glass transition (T_g) around −20 °C. Above 100 °C, the first run of both experiments showed a small peak in the curve, however, this did not occur for the second run. These small peaks may be the result of a correcting relaxation process to compensate for any uniformities that occurred during the synthesis. Unfortunately, the DSC curves could not indicate further differences between the phase-separated and non-phase-separated materials.

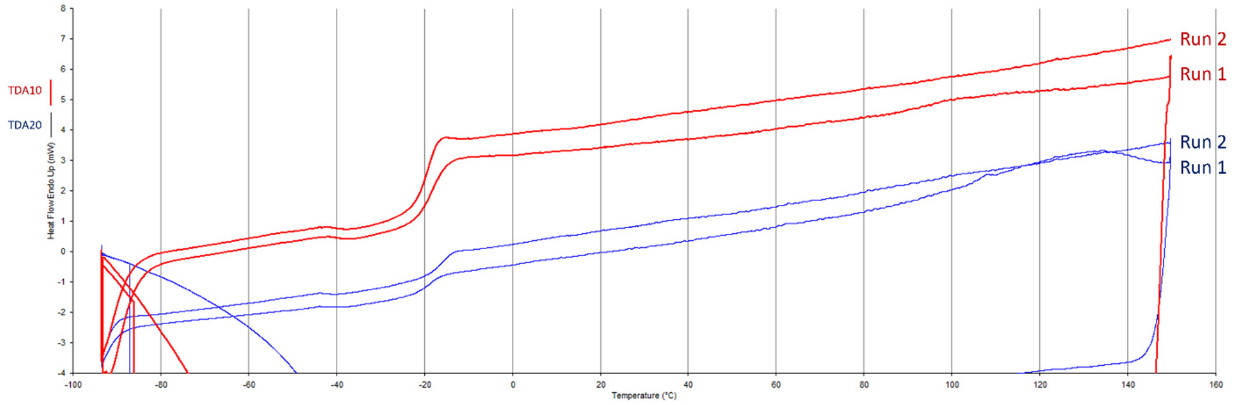

Figure S30. DSC curves of **P-TDA 10** (blue) and **P-TDA 20** (red).

16. Brightfield and Raman images

16.1 **P-ODA** samples

The Brightfield (top row) and Raman (bottom row) images of **P-ODA** samples from 5–12.5% **ODA** content (left to right columns respectively) are shown in Figure S31. The images show that phase separation occurred at/above 7.5% **ODA** content. Also, the **ODA+TA** domains (in yellow) have a slither-like appearance.

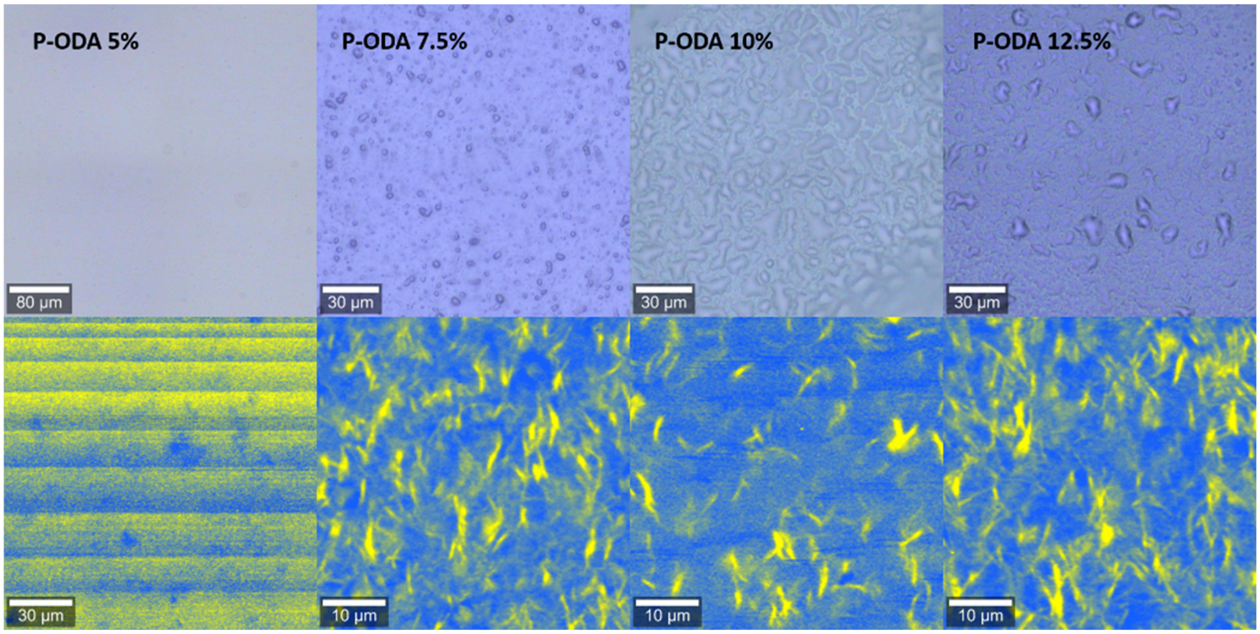

Figure S31. Brightfield (top row) and Raman (bottom row) images of **P-ODA** samples with 5 to 12.5% dianiline (**ODA**) contents (from right to left columns, respectively).

16.2 **P-KDA** samples

The Brightfield (top row) and Raman (bottom row) images of **P-KDA** with dianiline contents of 12.5% (left column) and 15% (right column) are shown in Figure S32. The Brightfield images do not show any indications of potential phase separation, but the Raman images show clearly that a phase separation occurred for **P-KDA 15**, while it did not for **P-KDA 12.5**.

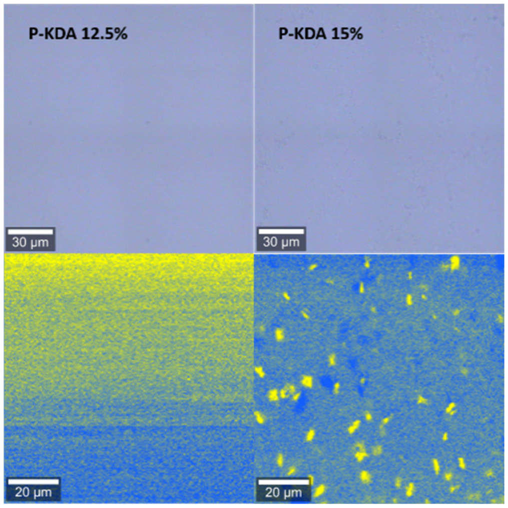

Figure S32. Brightfield (top row) and Raman (bottom row) images of **P-KDA 12.5** (left column) and **P-KDA 15** (right column).

16.3 **P-FDA** samples

The Brightfield (top row) and Raman (bottom row) images of **P-FDA** samples from 20 to 50% dianiline content are pictured in Figure S33 (From left to right: 20, 30, 40 and 50%). None of the obtained images show a noticeable phase separation. The Brightfield images of the **P-FDA 30** sample do show some noticeable features, however, the Raman could not elucidate any difference in chemical footprint. Therefore, the features seen in the Brightfield image are expected to be inhomogeneities of the surface. These possibly may have occurred during the drying process, for example by escaping air bubbles.

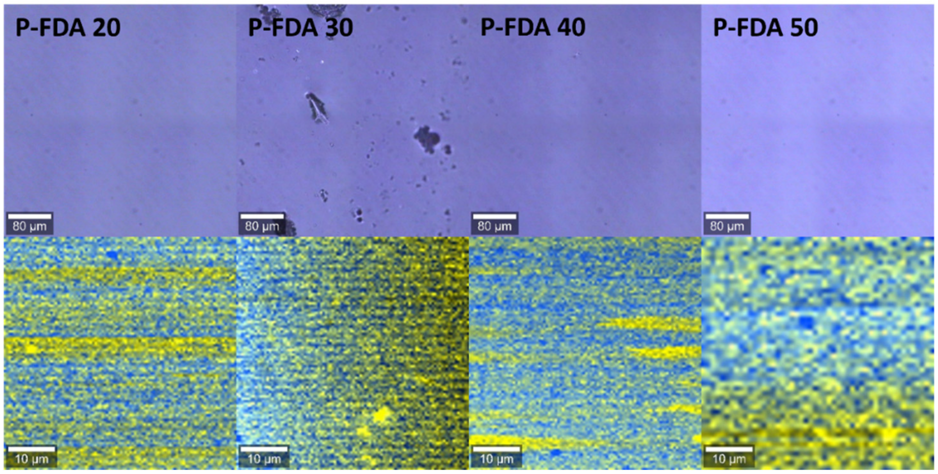

Figure S33. Brightfield (top row) and Raman (bottom row) images of **P-FDA** samples with dianiline (**FDA**) contents from 20 to 50% (left to right columns, respectively).

16.4 **P-SDA** samples

The Brightfield (top row) and Raman (bottom row) images of **P-SDA** with dianiline contents of 10 to 50% are pictured in Figure S34 (from left to right: 10, 20, 30, 40 and 50%). The **P-SDA** samples from 10 till 40% do not show any indication of phase separation in either the Brightfield or Raman images. The sample of **P-SDA 50** did have a distinct Brightfield image with a clear surface pattern. The Raman image was however not able to prove the existence of a phase separation, as the chemical footprint was similar over the entirety of the scanned area. This was rather unexpected as the physical appearance of the **P-SDA 50** sample was also glassy and turbid, whereas the **P-SDA 10-40** samples all appeared transparent and rubbery to gel-like (see pictures below). Potentially, due to the high dianiline *versus* aliphatic ratio, the rigidity of the polymer chains, as well as the crosslinking density, is increased, and as a result the materials may become brittle.

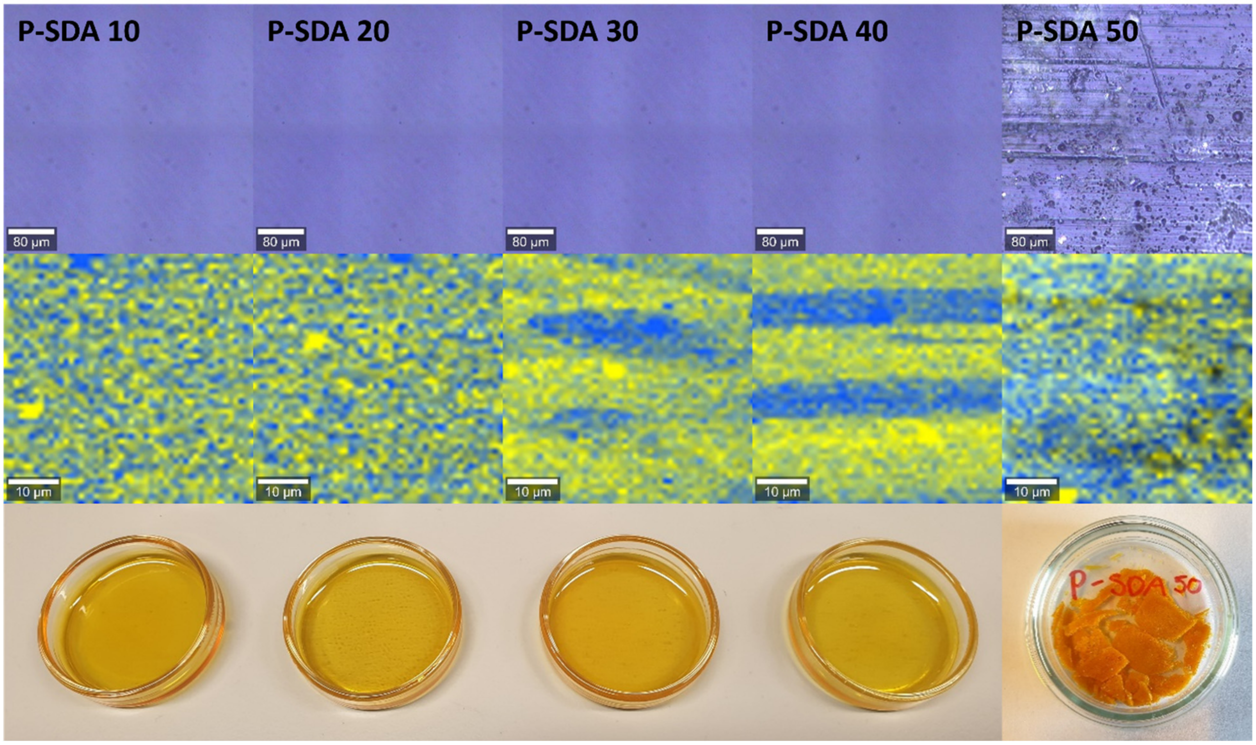

Figure S34. Brightfield images (top row), Raman images (middle row), and photos (bottom row) of the **P-SDA** materials with dianiline (**SDA**) contents from 10 to 50% (left to right columns, respectively).

16.5 **P-EDA** samples

Brightfield (top row) and Raman (bottom row) images of **P-EDA** materials with dianiline contents of 20%, 30% and 40% (from left to right column respectively) are shown in Figure S35 below. A (partial) phase separation was noted for all materials. See the main text for the continuous-scale Raman images. A photo of **P-EDA 20** is shown in Figure S36 that shows that the material also appears turbid, like all other phase-separated materials.

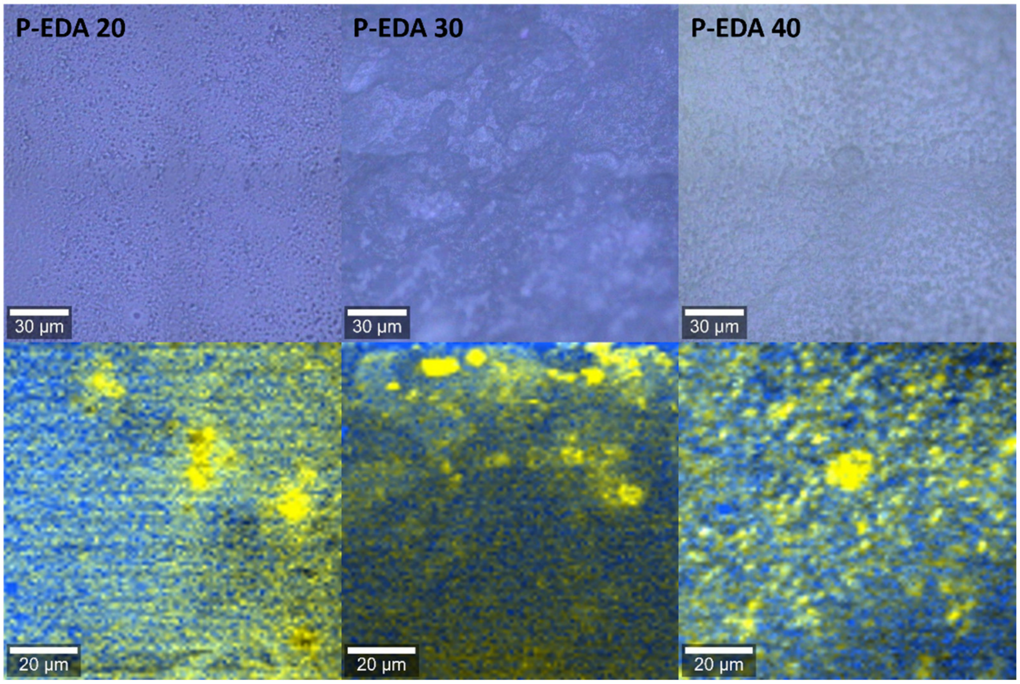

Figure S35. Brightfield (top row) and Raman (bottom row) images of **P-EDA** materials with dianiline (**EDA**) contents of 20, 30 and 40% (right to left columns, respectively).

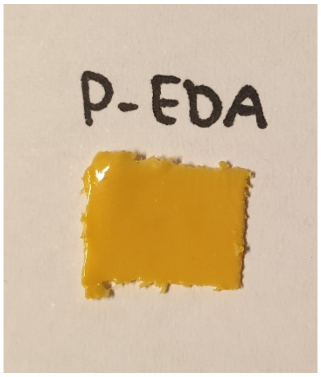

Figure S36. Photo of **P-EDA** material. The polymer film is turbid, which is typical for phase-separated materials.

17. Reversing phase separation

The reverse-phase-separation experiment was performed by starting from a phase-separated **P-TDA 20** film, and "diluting" this to a non-phase-separated **P-TDA 10** film. To do this, the **P-TDA 20** was swollen in chloroform, to which **TOTDDA**, **TREN**, and **TA** were added to decrease the overall dianiline content from 20% to 10%. This was done as follows:

First, we determined the molar mass of **P-TDA 20**, and defined 1 mole of **P-TDA 20** to consist of:

- 0.200 mole **TDA** (mw. 216.30 g·mol^{-1})
- 0.050 mole **TREN** (mw. 146.23 g·mol^{-1})
- 0.725 mole **TOTDDA** (mw. 220.31 g·mol^{-1})
- 1.000 mole **TA** (mw. 134.13 g·mol^{-1})

During the synthesis also 2 mole of H_2O (mw. 18.02 g·mol^{-1}) is lost.

Using the molar masses of the monomers, the total mass of 1 mole of **P-TDA 20** then becomes:

$$0.200 * 216.30 + 0.050 * 146.23 + 0.725 * 220.31 + 1.000 * 134.13 - 2 * 18.02 = \mathbf{308.4\ g}$$

We then started the experiment with 308.4 mg of **P-TDA 20**; representing exactly 1 mmol. To dilute from 20% to 10% **P-TDA** we thus need to add 0.050 mmol (7.5 µL) **TREN**, 0.925 mmol (203 µL) **TOTDDA** and 1.00 mmol (134.1 mg) **TA**.

First, a solution was prepared of **TREN** and **TOTDDA** in 5 mL chloroform, which was added to the in chloroform swollen **P-TDA 20** material (308 mg in 10 mL chloroform). The mixture was shaken thoroughly and left for 30 min at room temperature. Then, a solution of **TA** in 5 mL chloroform was prepared and added to the rest of the materials. The separate addition of amines and aldehyde monomers was done to prevent premature polymerization of **TA** with **TREN** and **TOTDDA** before addition to the swollen **P-TDA 20** film, or that the **P-TDA 20** film simply became "coated" with a layer of other polymer material. Once all components were added, the mixture was left for overnight at room temperature. The full content of the vial was then poured into a petri dish, and was left open to air for overnight so that the solvent could evaporate and a new polymer film formed. The material was further dried in a vacuum oven at 50 °C for 24 hours. The dried film was taken up for analysis to show that the phase separation had vanished.

18. References

S1. Schoustra, S. K.; Dijksman, J. A.; Zuilhof, H.; Smulders, M. M. J., Molecular control over vitrimer-like mechanics – tuneable dynamic motifs based on the Hammett equation in polyimine materials. *Chem. Sci.* **2021,** *12* (1), 293-302.

S2. Smit, C.; Swaaij, R. A. C. M. M. v.; Donker, H.; Petit, A. M. H. N.; Kessels, W. M. M.; Sanden, M. C. M. v. d., Determining the material structure of microcrystalline silicon from Raman spectra. *J. Appl. Phys.* **2003,** *94* (5), 3582-3588.

S3. Nandi, R.; Singh, H. K.; Singh, S. K.; Rao, D. S. S.; Prasad, S. K.; Singh, B.; Singh, R. K., Investigation of liquid crystalline property of a new calamitic liquid crystalline system methyl 4-(4′-(4″-(decyloxy)benzyloxy) benzylideneamino)benzoate. *Liq. Cryst.* **2017,** *44* (7), 1185-1193.

S4. John, N. L.; Joy, L. K.; Kumar, M. S.; Shaiju, S. S.; Subashini, A.; Sajan, D., Quantitative structure and activity relationship on the biological, nonlinear and the spectroscopic properties of the Schiff base material: 4-chloro-4′bromobenzylidene aniline. *Mol. Simul.* **2018,** *44* (1), 40-54.

S5. Lee, M.; Lee, J.-P.; Rhee, H.; Choo, J.; Gyu Chai, Y.; Kyu Lee, E., Applicability of laser-induced Raman microscopy for in situ monitoring of imine formation in a glass microfluidic chip. *J. Raman Spectrosc.* **2003,** *34* (10), 737-742.

S6. Knöpke, L. R.; Nemati, N.; Köckritz, A.; Brückner, A.; Bentrup, U., Reaction Monitoring of Heterogeneously Catalyzed Hydrogenation of Imines by Coupled ATR-FTIR, UV/Vis, and Raman Spectroscopy. *ChemCatChem* **2010,** *2* (3), 273-280.

S7. Badawi, H. M., A comparative study of the structure and vibrational spectra of diphenylmethane, the carcinogen 4,4'-methylenedianiline and 4,4'-methylenebis(N,N-dimethylaniline). *Spectrochim. Acta A Mol. Biomol. Spectrosc.* **2013,** *109*, 213-20.

S8. Ullah, R.; Wang, X., Raman spectroscopy of Bisphenol 'S' and its analogy with Bisphenol 'A' uncovered with a dimensionality reduction technique. *J. Mol. Struct.* **2019,** *1175*, 927-934.
